# Supplementary material for: Amazon River plume habitats shape planktonic cnidarian assemblages in the Western Atlantic
Source: PLoS One. 2023 Aug 25;18(8):e0290667. doi: 10.1371/journal.pone.0290667 (PMC10456164; doi:10.1371/journal.pone.0290667)

PLOS ONE

Amazon River plume habitats shape planktonic cnidarian assemblages in the Western Atlantic

--Manuscript Draft--

**Manuscript Number:**	PONE-D-23-10398
**Article Type:**	Research Article
**Full Title:**	Amazon River plume habitats shape planktonic cnidarian assemblages in the Western Atlantic
**Short Title:**	Amazon River plume habitats shape planktonic cnidarian assemblages in the Western Atlantic
**Corresponding Author:**	Everton Giachini Tosetto<br>Universidade Federal de Pernambuco<br>Recife, Pernambuco BRAZIL
**Keywords:**	Jellyfish; Medusae; Siphonophores; Zooplankton; North Brazil Current
**Abstract:**	The impact of the Amazon River freshwater plume on planktonic cnidarians over neritic and oceanic provinces is unknown. To provide further knowledge we took advantage of an oceanographic cruise performed in October 2012 in the Western Atlantic off the North Brazilian coast (8°N, 51°W - 3.5°S, 37°W). A complex and dynamic system was observed, strong currents and eddies dispersing the plume over a large area. Our results show that the freshwater, nutrients and sediment runoff of the Amazon River shapes a three-dimensional habitat with a thin highly productive surface layer compressed by a deeper oxygen minimum zone both over the shelf and in the open ocean. We concluded that such habitat structure is particularly advantageous to planktonic cnidarians, which have low metabolic rates, being able to survive in hypoxic zones, resulting in high species richness and abundance. Over the shelf, distinctions were sharp and the area under the influence of the plume presented a diverse assemblage occurring in large abundance. Persa incolorata, Enneagonun hyalinun, Muggiaea kochii and Diphyes dispar were representative species in this system, while outside the plume, the hydromedusa Liriope tetraphylla was dominant and occurred almost alone. Divergences in the oceanic province were less pronounced but still expressive. Chelophyes appendiculata, Bassia bassensis, Eudoxoides spiralis and Abylopsis tetragona were more abundant outside the plume while Diphies bojani, Eudoxoides mitra, D. dispar and Nanomia bijuga were more abundant inside. We conclude that Amazon River plume is a paramount physical feature shaping planktonic cnidarian assemblages both in neritic and oceanic provinces.
**Order of Authors:**	Everton Giachini Tosetto
	Sigrid Neumann-Leitão
	Moacyr Araujo
	Djoirka Minto Dimoune
	Arnaud Bertrand
	Miodeli Nogueira Júnior
**Additional Information:**	
**Question**	**Response**
**Financial Disclosure**<br><br>Enter a financial disclosure statement that describes the sources of funding for the work included in this submission. Review the submission guidelines for detailed requirements. View published research	The author received no specific funding for this work.

articles from *PLOS ONE* for specific examples.<br><br>This statement is required for submission and **will appear in the published article** if the submission is accepted. Please make sure it is accurate.<br><br>**Unfunded studies**<br>Enter: *The author(s) received no specific funding for this work.*<br><br>**Funded studies**<br>Enter a statement with the following details:<br>• Initials of the authors who received each award<br>• Grant numbers awarded to each author<br>• The full name of each funder<br>• URL of each funder website<br>• Did the sponsors or funders play any role in the study design, data collection and analysis, decision to publish, or preparation of the manuscript?<br>• **NO** - Include this sentence at the end of your statement: *The funders had no role in study design, data collection and analysis, decision to publish, or preparation of the manuscript.*<br>• **YES** - Specify the role(s) played.<br><br>* typeset	
**Competing Interests**<br><br>Use the instructions below to enter a competing interest statement for this submission. On behalf of all authors, disclose any competing interests that could be perceived to bias this work—acknowledging all financial support and any other relevant financial or non-financial competing interests.<br><br>This statement is required for submission and **will appear in the published article** if the submission is accepted. Please make sure it is accurate and that any funding sources listed in your Funding Information later in the submission form are also declared in your Financial Disclosure statement.	The author(s) received no specific funding for this work.

View published research articles from *PLOS ONE* for specific examples.<br><br>**NO authors have competing interests**<br><br>Enter: *The authors have declared that no competing interests exist.*<br><br>**Authors with competing interests**<br><br>Enter competing interest details beginning with this statement:<br><br>*I have read the journal's policy and the authors of this manuscript have the following competing interests: [insert competing interests here]*<br><br>* typeset	
**Ethics Statement**<br><br>Enter an ethics statement for this submission. This statement is required if the study involved:<br><br>• Human participants<br>• Human specimens or tissue<br>• Vertebrate animals or cephalopods<br>• Vertebrate embryos or tissues<br>• Field research<br><br>Write "N/A" if the submission does not require an ethics statement.<br><br>General guidance is provided below. Consult the submission guidelines for detailed instructions. **Make sure that all information entered here is included in the Methods section of the manuscript.**	N/A

**Format for specific study types**<br><br>**Human Subject Research (involving human participants and/or tissue)**<br>• Give the name of the institutional review board or ethics committee that approved the study<br>• Include the approval number and/or a statement indicating approval of this research<br>• Indicate the form of consent obtained (written/oral) or the reason that consent was not obtained (e.g. the data were analyzed anonymously)<br><br>**Animal Research (involving vertebrate animals, embryos or tissues)**<br>• Provide the name of the Institutional Animal Care and Use Committee (IACUC) or other relevant ethics board that reviewed the study protocol, and indicate whether they approved this research or granted a formal waiver of ethical approval<br>• Include an approval number if one was obtained<br>• If the study involved *non-human primates*, add *additional details* about animal welfare and steps taken to ameliorate suffering<br>• If anesthesia, euthanasia, or any kind of animal sacrifice is part of the study, include briefly which substances and/or methods were applied<br><br>**Field Research**<br><br>Include the following details if this study involves the collection of plant, animal, or other materials from a natural setting:<br>• Field permit number<br>• Name of the institution or relevant body that granted permission	
**Data Availability**<br><br>Authors are required to make all data underlying the findings described fully available, without restriction, and from the time of publication. PLOS allows rare exceptions to address legal and ethical concerns. See the PLOS Data Policy and FAQ for detailed information.	Yes - all data are fully available without restriction

A Data Availability Statement describing where the data can be found is required at submission. Your answers to this question constitute the Data Availability Statement and **will be published in the article**, if accepted.<br><br>**Important:** Stating 'data available on request from the author' is not sufficient. If your data are only available upon request, select 'No' for the first question and explain your exceptional situation in the text box.<br><br>Do the authors confirm that all data underlying the findings described in their manuscript are fully available without restriction?	
**Describe where the data may be found in full sentences. If you are copying our sample text, replace any instances of XXX with the appropriate details.**<br><br>• If the data are **held or will be held in a public repository**, include URLs, accession numbers or DOIs. If this information will only be available after acceptance, indicate this by ticking the box below. For example: *All XXX files are available from the XXX database (accession number(s) XXX, XXX.)*.<br>• If the data are all contained **within the manuscript and/or Supporting Information files**, enter the following: *All relevant data are within the manuscript and its Supporting Information files.*<br>• If neither of these applies but you are able to provide **details of access elsewhere**, with or without limitations, please do so. For example:<br><br>*Data cannot be shared publicly because of [XXX]. Data are available from the XXX Institutional Data Access / Ethics Committee (contact via XXX) for researchers who meet the criteria for access to confidential data.*<br><br>*The data underlying the results presented in the study are available from (include the name of the third party*	The data underlying the results presented in the study are available in SEANOAE (https://doi.org/10.17882/93375).

and contact information or URL).<br>• This text is appropriate if the data are owned by a third party and authors do not have permission to share the data.<br><br>* typeset	
Additional data availability information:	

Powered by Editorial Manager® and ProduXion Manager® from Aries Systems Corporation

Manuscript
Click here to access/download;Manuscript;Main text planktonic cnidarians ARP PLOS.docx

Amazon River plume habitats shape planktonic cnidarian assemblages in the Western Atlantic

Everton Giachini Tosetto[123], Sigrid Neumann-Leitão[3], Moacyr Araujo[34], Djoirka Minto Dimoune[3], Arnaud Bertrand[1235], Miodeli Nogueira Júnior[6]

[1] Institut de Recherche pour le Développement, Sète, 34200, France.

[2] MARBEC, Univ Montpellier, CNRS, IFREMER, IRD, Sète, 34200, France.

[3] Departamento de Oceanografia, Universidade Federal de Pernambuco, Recife, 50670-901, Brazil.

[4] Brazilian Research Network on Global Climate Change (Rede CLIMA), São José dos Campos, 12227-010, Brazil.

[5] Departamento de Pesca e Aquicultura, Universidade Federal Rural de Pernambuco, Recife, 52171-900, Brazil.

[6] Departamento de Sistemática e Ecologia, Universidade Federal da Paraíba, João Pessoa, 58051-900, Brazil.

Correspondence to: Everton Giachini Tosetto (evertontosetto@hotmail.com), Arnaud Bertrand (arnaud.bertrand@ird.fr) and Miodeli Nogueira Júnior (miodeli@gmail.com).

Abstract

The impact of the Amazon River freshwater plume on planktonic cnidarians over neritic and oceanic provinces is unknown. To provide further knowledge we took advantage of an oceanographic cruise performed in October 2012 in the Western Atlantic off the North Brazilian coast (8°N, 51°W - 3.5°S, 37°W). A complex and dynamic system was observed, strong currents and eddies dispersing the plume over a large area. Our results show that the freshwater, nutrients and sediment runoff of the Amazon River shapes a three-dimensional habitat with a thin highly productive surface layer compressed by a deeper oxygen minimum zone both over the shelf and in the open ocean. We concluded that such habitat structure is particularly advantageous to planktonic cnidarians, which have low metabolic rates, being able to survive in hypoxic zones, resulting in high species richness and abundance. Over the shelf, distinctions were sharp and the area under the influence of the plume presented a diverse assemblage occurring in large abundance. *Persa incolorata, Enneagonun hyalinun, Muggiaea kochii* and *Diphyes dispar* were representative species in this system, while outside the plume, the hydromedusa *Liriope tetraphylla* was dominant and occurred almost alone. Divergences in the oceanic province were less pronounced but still expressive. *Chelophyes appendiculata, Bassia bassensis, Eudoxoides spiralis* and *Abylopsis tetragona* were more abundant outside the plume while *Diphies bojani*, *Eudoxoides mitra*, *D. dispar* and *Nanomia bijuga* were more abundant inside. We conclude that Amazon River plume is a paramount physical feature shaping planktonic cnidarian assemblages both in neritic and oceanic provinces.

Keywords. Jellyfish, Medusae, Siphonophores, Zooplankton, North Brazil Current

1. Introduction

In marine environments, planktonic cnidarians respond to changes in physical and biogeochemical environments over a wide range of spatiotemporal scales. Global cnidarian diversity, distribution and abundance patterns are closely related to oceanographic dynamics

and water masses, and to climate patterns [1–3]. Currents, eddies, fronts of water masses, upwelling and other physical processes can drive their distribution at regional scales [1,4–9]. Finally, responses to local changes in the environment, such as prey availability and water temperature and salinity, may determine species local occurrence and abundance [10–12].

In the past, planktonic cnidarians were often set aside in traditional zooplankton studies. However, their high feeding rates and significant role as predators in the trophic web, associated with large population blooms, which occur in the life cycles of many species, have the potential to control the pelagic community and collapse fisheries [13,14]. Therefore, interest in pelagic cnidarian ecology, their responses to environmental conditions, such as bloom trigger mechanisms, and their role in ecosystem functioning as predators and prey for higher tropic levels increased in recent decades [14–17]. However, knowledge on the ecology of planktonic cnidarians is still scarce in low latitude systems such as the Western Equatorial Atlantic Ocean.

As a western boundary current system, a strong current, the North Brazil Current (NBC) flows coastward in this region [18,19]. Such feature results in intrusions of oligotrophic oceanic waters and associated planktonic cnidarian fauna over the continental shelf in other western boundary systems [20,21]. Despite the oligotrophic scenario brought by the NBC, the Amazon River discharges up to 2.4×10^5 m^3s^{-1} of freshwater (approximately 20% of global freshwater run-off) with organic matter, nutrients and sediments over the area [22,23]. The Amazon discharge creates a surface plume (Amazon River plume, hereafter ARP) of low-salinity, high nutrients, and suspended and dissolved materials that reaches thousands of kilometers in the Equatorial and North Atlantic and Caribbean Sea [24–26] with strong influence on ecological processes. The large discharge of nutrients enhances primary production and phytoplankton concentration and possibly the whole marine community by a bottom-up effect, as observed with planktonic decapods [27,28]. The low salinity brackish environment may affect the spatial distribution of marine animals such as planktonic cnidarians, which usually are associated with specific environmental saline conditions and water masses [5,9,29,30]. Additionally, bellow the

plume, low oxygen levels are observed due the high rates of sinking organic matter mineralization [31,32]

Freshwater run-off from small rivers are known to affect the structure of planktonic cnidarian assemblage in coastal areas [33–39] where species have distinct responses to the salinity gradient. In the Western Equatorial Atlantic, the ARP extends far offshore and its influence on planktonic cnidarian distribution and abundance may occur thousands of kilometers away from the river mouth. However, effects of such major river plumes on cnidarians were never specifically evaluated. In this study, we investigate the hypothesis according to which unlike other western boundary current systems, the ecological processes induced by the spread of the ARP, drive the structure of planktonic cnidarian assemblages both over the shelf and offshore. For that purpose, we used a comprehensive set of data collected from neritic and oceanic provinces in the Western Equatorial Atlantic, both inside and outside the influence of the ARP, in order to understand the response of the planktonic cnidarian assemblage to this unique physical and biogeochemical environment.

2. Materials and Methods

2.1. Study area

The study area, along the North Brazilian continental shelf between the Amazon and Oyapok river mouths and equatorial Atlantic oceanic waters, ranges between 8°N, 51°W and 3.5°S, 37°W (Fig. 1). The continental shelf reaches up to 300 km wide and the shelf break occurs around 120 m depth [40,41]. The large freshwater discharge of the Amazon River creates an extensive surface plume. Strong ocean currents, eddies, wind fields and high tidal variation in the North Brazilian continental shelf and adjacent oceanic waters shape the dispersion of the ARP resulting in a highly dynamic system with high spatial and temporal variability [41–44]. Three general patterns occur in the spread of the ARP throughout the year. From January to April, the ARP flows continuously northwest along the Brazilian coast carried mainly by NE winds.

From April to July, the ARP reaches the Caribbean region due to the higher discharge, NBC transport and SE winds. Finally, from August to December, when wind fields are weaker, the retroflection of the NBC, around 5 and 10°N, disperses the ARP to the east, feeding the North Equatorial Counter Current [NECC; 43,45]. During this period the plume that can exceed 10^6 km^2, reaching longitudes to the east as far as 25°W [46].

Figure 1. Geographic location of the study area in the North Brazilian continental shelf and adjacent Western Equatorial Atlantic Ocean, showing the sampled stations.

2.2. Sampling

Data were obtained during the oceanographic cruise *Camadas Finas* III, aboard the research vessel NHo. Cruzeiro do Sul - H38 (DHN/Brazilian Navy). It was performed in 9-31 October 2012, the period when most of the ARP is retroflexed and transported eastward by the NECC [43,45]. Zooplankton was sampled at 44 stations in oblique hauls, using Bongo nets with 120 and 300 µm mesh and 0.3 and 0.6 m mouth diameter, respectively. Tows were performed at approximately 2 knots from near bottom to surface on the continental shelf, and from 200 m to the surface in the open ocean. Nets were fitted with a flowmeter (Hydro-Bios) to estimate the filtered volume . Samples were fixed with 4% formaldehyde buffered with sodium tetraborate (0.5 gL^{-1}). Current speed and directions were recorded along all the track of the ship by a Teledyne RD Ocean Surveyor ADCP. Data from the first 100 m of the water column were integrated each 30 km along the track. Salinity, temperature (°C), density (σ_t), dissolved oxygen (mgL^{-1}) and fluorescence vertical profiles were achieved at stations where zooplankton was sampled with a Seabird SBE 25 Sealogger CTD profiler [47].

In laboratory, whole zooplankton samples were analyzed under stereomicroscope and specimens were identified [mainly following 48,49] and counted. A general taxonomic overview of planktonic cnidarians of the area was previously provided [50]. Abundances were

standardized in number of individuals per 100 m^{-3} for medusae and number of colonies per 100 m^{-3} for siphonophores[illegible]or calycophorans, the number of anterior nectophores was used for estimating the polygastric stage abundance, and eudoxid bracts for the eudoxid stage abundance [e.g. 30,5[illegible]or physonects and the calycophoran *Hippopodius hippopus*, number of colonies were roughly estimated by dividing the number of nectophores by 10 [52[illegible]et weight from 120 µm mesh samples (including cnidarians) measured by gravimetry after removing the excess water with blotted paper [53] were used as an indirect estimator zooplankton biomass (mostly copepods, cladocerans and other crustaceans).

2.3. Data analysis

The ARP was delimited by the isohaline of 35 and density <22. Tropical Surface Water (TSW) and South Atlantic Central Water (SACW) masses were delimited by the isobar of 24.5 [43,54,55]. Cyclonic and anticyclonic eddies were respectively identified by negative and positive sea levels anomalies in daily L4 satellite data, measured by multi-satellite altimetry observations over Global Ocean, produced by SSALTO/DUACS and distributed by European Union Copernicus Marine Service Information. Dominant current in each station was determined by the overall direction observed in the ADCP data and classified as NBC, retroflection area (RETRO) and NECC.

The abundance, species richness and structure of the planktonic cnidarian assemblage was statistically similar in 120 and 300 µm meshes [56]. Thus, we merged both meshes for data analysis. Station 1, the was the unique spot sampled inside the river estuary and was excluded from the data analysis. For statistical tests, abundance data was transformed by log (x+1). Analyses of Variance (ANOVA) were performed to test for differences in abundance of the most abundant planktonic cnidarian species, according to the province (neritic and oceanic) and influence of the ARP. Tukey post-hoc test was used to identify the domains that differed when ANOVA was significant. Spatial patterns in planktonic cnidarian assemblage abundance were identified by a hierarchical cluster analysis (Bray-Curtis similarity matrix). A Similarity Percentage

(SIMPER) analysis was performed in order to identify key species and their contribution to similarity within the groups defined in the cluster analysis.

To identify associations between representative planktonic cnidarian taxa (species occurring in more than 21 stations and species with high abundance in few stations) and the physical environment, a constrained ordination analysis was performed. Detrended Canonical Correspondence Analysis (DCCA) revealed a small length of environmental gradients (<3), indicating that a linear method was appropriate, and thus Redundancy Analysis (RDA) was selected [57]. Mesoscale physical processes were included as dummy categorical explanatory variables (neritic and oceanic habitats, presence of ARP, predominant current, presence of cyclonic and anticyclonic eddies). 120 µm mesh zooplankton biomass (considered as food availability), maximum value of fluorescence (as a proxy of biological productivity), maximum value of dissolved oxygen and surface temperature and salinity were included as continuous explanatory variables. Monte Carlo test was used to test the significance of the first and all canonical axes together [57].

Environmental and biological distribution maps were produced in Ocean Data View 5.0 and Qgis 3.2.1. Cluster and SIMPER analysis were performed in Primer v.6 + PERMANOVA. DCCA and RDA were performed in CANOCO 4.5.

3. Results

3.1. Environmental background

The southern part of the studied area (reaching 3°N) was dominated by the North Brazilian Current (NBC), flowing west and coastwards. Around 5°N (station 10), the NBC was retroflexed northwards, where faster currents occurred. In the north of the study area, from 7.5°N, 46°W (station 15) to 4°N, 38°W (station 28) the North Equatorial Counter Current (NECC), flowing east, predominated (Fig. 2a). One cyclonic eddy, causing surface divergence, occurred near the mouth of the Amazon River, stations 4, 5 and 6 were sampled under its influence.

Stations 8, 13, 14, 17, 18 were sampled under the influence of three anticyclonic eddies and surface convergence (Fig. 2b). Although another cyclonic eddy occurred in the west side of the study area, it was already dissipated in the day those stations were sampled.

Figure 2. (a) Surface currents vectors and indicators of the predominant current in the area (NBC = North Brazilian Current; RETRO = North Brazilian Current retroflection; NECC=North Equatorial Counter Current). **(b)** Sea level anomalies and indicators of cyclonic and an anticyclonic eddies. **(c)** Sea surface salinity and estimated position of 35 isohaline delimitating the Amazon River plume (ARP) and tropical surface water (TSW). **(d)** Sea surface temperature. **(e)** Surface density **(f)** Surface dissolved oxygen. **(g)** Surface fluorescence. **(h)** Total zooplankton biomass found in the first 200 m of the water column (mean of 120 and 300 µm meshes). All data from October 2012.

Over the continental shelf, surface waters of stations 8 and 9 were influenced by the ARP resulting in salinities below 35 in the first 8 m of the water column (Fig. 2c; 3a). In the oceanic province, stations 15 to 24 were influenced by the ARP, where the 35 isohaline ranged from 14 m depth at station 21 to 59 m depth at station 19. Outside the ARP, surface salinity was around 36 in all stations (Fig. 2c).

Figure 3. Vertical profiles and contours of **(a)** Salinity, traced line is the position of 35 isohaline delimitating the Amazon River plume. **(b)** Temperature. **(c)** Density. **(d)** Fluorescence. **(e)** Dissolved oxygen. Upper bars indicate the main mesoscale processes observed in the area. All data from October 2012.

Coastal surface waters were slightly warmer than adjacent oceanic areas, reaching 28.5°C. In oceanic waters, surface temperature oscillated mainly with latitude with higher

temperatures in north of the study area, reaching 29.8°C (Fig. 2d). Colder waters (<18°C) occurred commonly around 120 m depth, below a strong thermocline, however intrusions in the upper layers were observed at stations 21 to 24 were it reached up to 60 m depth. Distinctly, close to the continental slope, warm surface water transposed down to 150 m depth (Fig. 3b). These upwelling and downwelling features reflect the complex circulation system in the area. Following the temperature and salinity gradients, three water masses were observed in the first 200 m, ARP waters (σ_T < 22), Tropical Surface Waters (TSW) (σ_T between 22 and 24.5) and South Atlantic Central Waters (SACW) (σ_T > 24.5; Fig. 2e; 3c).

Florescence was higher in the surface layer of neritic stations influenced by the ARP, reaching 2.7 (Fig. 2g). A small increment in fluorescence was also observed near the bottom at station 5, which was under the influence of a cold core cyclonic eddy. In the open ocean, a deep fluorescence maximum layer was observed in the boundary between TSW and SACW (Fig. 3d). High zooplankton biomass occurred in the neritic area under influence of the ARP and in the region of the cold core cyclonic eddy, no clear spatial patterns were observed in other stations (Fig. 2h). Dissolved oxygen was steady in the surface layer over the entire study area. In deeper waters, both in neritic and oceanic zones under the influence of the ARP, an oxygen minimum layer (<3 $mg.l^{-1}$) was observed between 50 and 200 m depth (Fig. 2f; 3e).

3.2 Species composition

A total of 91 taxa of planktonic cnidarians were collected (Table 1), corresponding to 2 scyphomedusae, 41 hydromedusae and 48 siphonophores. Furthermore, many unidentified cerinula, ephyrae and athorybia larval forms were collected. *Liriope tetraphylla* was the most frequent medusa, being present in 88.5% of the samples all over the study area, followed by *Aglaura hemistoma* (78.2%) and *Sminthea eurygaster* (41.4%). Among siphonophores, the most frequent were *Diphyes bojani* (88.5%), *Bassia bassensis* (80.5%), *Chelophyes appendiculata*

Type text here

(78.2%), *Abylopsis tetragon*a (75.9%), *Nanomia bijuga* (74.7%) and *Eudoxoides mitra* (75.6%; Table 1).

Table 1. Basic statistics of planktonic cnidarian species from neritic and oceanic provinces in the Western Equatorial Atlantic Ocean off North Brazil. Mean abundance (ind. 100 m^{-3}) per station and standard deviation, range of abundance and frequency of occurrence (f; considering both provinces; n= 87 samples). Species are sorted by frequency of occurrence. In all cases, both meshes were pooled.

**Species**	**Neritic**		**Oceanic**		**f**
	**Mean ± SD**	**Range of non-zero abundances**	**Mean ± SD**	**Range of non-zero abundances**	
**Siphonophores**					
Diphyes bojani (Eschscholtz, 1825)	19.7 ± 61.4	0.6 - 263.3	50.6 ± 81.4	2.8 - 471.4	88.5
Bassia bassensis (Quoy & Gaimard, 1833)	3.1 ± 8.33	1.6 - 30.5	19.7 ± 14.3	1.8 - 73.09	80.5
Chelophyes appendiculata (Eschscholtz, 1829)	8.6 ± 33.4	22.9 - 148.8	37.7 ± 35.6	3 - 161.9	78.1
Abylopsis tetragona (Otto, 1823)	2.1 ± 5.98	0.8 - 22.9	8.6 ± 8.3	0.9 - 40.5	75.9
Nanomia bijuga (Delle Chiaje, 1844)	3.3 ± 6.85	0.9 - 28.7	2.5 ± 2.9	0.1 - 13.5	74.7
Eudoxoides mitra (Huxley, 1859)	1 ± 2.94	0.8 - 11.4	34.1 ± 40.1	0.3 - 214.6	73.6
Abylopsis eschscholtzii (Huxley, 1859)	1.9 ± 7.67	2.9 - 34.3	7 ± 12.3	0.3 - 92.7	69
Sulculeolaria chuni (Lens & van Riemsdijk, 1908)	7.4 ± 28.35	22.9 - 125.9	7.6 ± 8.3	0.2 - 33.4	69
Eudoxoides spiralis (Bigelow, 1911)	1.7 ± 7.68	34.3 - 34.3	13.3 ± 21.3	0.7 - 123.1	60.9
Diphyes dispar Chamisso & Eysenhardt, 1821	72.1 ± 187.61	0.7 - 768.7	1.7 ± 2.9	0.3 - 15.2	57.5
Agalma okenii Eschscholtz, 1825	0.4 ± 1.71	7.6 - 7.6	1.1 ± 1.4	0.1 - 6.8	54
Sulculeolaria turgida (Gegenbaur, 1854)	-	-	0.9 ± 1.5	0.1 - 7	37.9
Ceratocymba leuckartii (Huxley, 1859)	-	-	1 ± 1.5	0.1 - 5.9	36.8
Lensia campanella (Moser, 1917)	0.4 ± 1.6	1.56 - 7.2	1 ± 1.7	0.3 - 7.6	34.5
Agalma elegans (Sars, 1846)	-	-	0.6 ± 1.2	0.3 - 6.7	29.9
Sulculeolaria biloba (Sars, 1846)	-	-	0.8 ± 1.4	0.4 - 5.4	29.9
Cordagalma ordinatum (Haeckel, 1888)	-	-	0.5 ± 0.9	0.3 - 3.9	27.6
Lensia meteori (Leloup, 1934)	-	-	1 ± 2.3	0.3 - 14.9	25.3
Lychnagalma utricularia (Claus, 1879)	0.7 ± 2.37	3.6 - 10.2	0.5 ± 1.2	0.5 - 7.4	25.3

Species	Neritic		Oceanic		f
	Mean ± SD	Range of non-zero abundances	Mean ± SD	Range of non-zero abundances	
Hippopodius hippopus (Forsskål, 1776)	-	-	0.3 ± 0.7	0.1 - 2.8	18.4
Amphicaryon sp.	-	-	0.3 ± 0.8	0.4 - 3.5	14.9
Halistemma rubrum (Vogt, 1852)	-	-	0.2 ± 0.5	0.4 - 1.9	14.9
Lensia subtilis (Chun, 1886)	-	-	0.2 ± 0.7	0.1 - 3.4	11.5
Lensia spp.	-	-	0.2 ± 0.8	0.6 - 4.6	8
Muggiaea kochii (Will, 1844)	95.7 ± 282.9	70.04 - 1185.4	0.2 ± 1.3	1.9 - 9.1	8
Athorybia rosacea (Forsskål, 1775)	0.1 ± 0.4	1.6 - 1.6	0.1 ± 0.7	0.4 - 4.7	6.9
Rosacea plicata Bigelow, 1911	-	-	0.1 ± 0.5	0.6 - 2.5	6.9
Sulculeolaria monoica (Chun, 1888)	-	-	0.1 ± 0.5	0.5 - 2.4	6.9
Enneagonum hyalinum Quoy & Gaimard, 1827	207.1 ± 589.1	152.8 - 2227.1	0.1 ± 0.2	1.4 - 1.4	5.7
Chuniphyes sp.	-	-	0.1 ± 0.5	0.1 - 4.2	4.6
Lensia cossack Totton, 1941	-	-	0.1 ± 0.1	0.4 - 0.6	4.6
Lensia fowleri (Bigelow, 1911)	-	-	0.1 ± 0.3	0.3 - 1.6	4.6
Abyla sp.	-	-	0.1 ± 0.2	0.1 - 1.1	3.4
Abyla trigona Quoy & Gaimard, 1827	-	-	0.1 ± 0.3	0.8 - 1.6	3.4
Forskalia edwardsii Kölliker, 1853	-	-	0.1 ± 0.3	1.2 - 1.5	3.4
Lensia conoidea (Keferstein & Ehlers, 1860)	-	-	0.1 ± 0.2	0.5 - 1.5	3.4
Lensia hardy Totton, 1941	-	-	0.1 ± 0.9	0.7 - 6.5	3.4
Sphaeronectes koellikeri Huxley, 1859	-	-	0.1 ± 0.3	0.9 - 1.9	3.4
Forskalia contorta (Milne Edwards, 1841)	-	-	0.1 ± 0.3	0.5 - 2.1	2.3
Lensia subtiloides (Lens & van Riemsdijk, 1908)	-	-	0.1 ± 0.1	0.3 - 0.3	2.3
Amphicaryon peltifera (Haeckel, 1888)	-	-	0.1 ± 0.28	2.3 - 2.3	1.1
Dimophyes arctica (Chun, 1897)	-	-	0.1 ± 0.76	6.2 - 6.2	1.1
Lensia hotspur Totton, 1941	-	-	0.1 ± 1	8.2 - 8.2	1.1

Species	Neritic		Oceanic		f
	Mean ± SD	Range of non-zero abundances	Mean ± SD	Range of non-zero abundances	
Lensia leloupi Totton, 1954	-	-	0.1 ± 0.2	1.9 - 1.9	1.1
Physophora hydrostatica Forsskål, 1775	-	-	0.1 ± 0.3	2.5 - 2.4	1.1
Rosacea sp.	-	-	0.1 ± 0.1	1.2 - 1.2	1.1
Sulculeolaria quadrivalvis de Blainville, 1830	-	-	0.1 ± 0.1	0.1 - 0.1	1.1
**Hydromedusae**					
Liriope tetraphylla (Chamisso & Eysenhardt, 1821)	322.1 ± 582.2	2.8 - 2199.6	9.2 ± 14.6	0.3 - 99.3	88.5
Aglaura hemistoma Péron & Lesueur, 1810	10.3 ± 31.3	11.6 - 125.9	33 ± 37.6	0.1 - 192.7	78.2
Sminthea eurygaster Gegenbaur, 1857	-	-	2.5 ± 4	0.2 - 16.7	41.4
Rhopalonema velatum Gegenbaur, 1857	0.6 ± 2.6	11.5 - 11.4	1.1 ± 1.9	0.1 - 7.9	35.6
Solmundella bitentaculata (Quoy & Gaimard, 1833)	0.6 ± 1.7	1.6 - 7.2	1.1 ± 2	0.5 - 9.3	34.5
Cytaeis sp.1	0.4 ± 1.7	7.6 - 7.6	0.5 ± 1	0.3 - 5.5	29.9
Annatiara affinis (Hartlaub, 1914)	0.4 ± 1.7	7.6 - 7.6	0.4 ± 1.5	0.6 - 10.6	12.6
Clytia spp.	0.5 ± 1.2	3.5 - 3.5	0.1 ± 0.4	0.5 - 1.9	11.5
Cunina octonaria McCrady, 1859	5.6 ± 17.2	4.8 - 75.4	0.1 ± 0.7	0.5 - 4.5	9.2
Cirrholovenia tetranema Kramp, 1959	2.5 ± 7.7	2.4 - 34.3	0.1 ± 0.5	1 - 3	8
Persa incolorata McCrady, 1859	222.7 ± 646.7	3.6 - 2701.3	0.2 ± 1.5	12 - 12.0	6.9
Eucheilota maculata Hartlaub, 1894	0.6 ± 2.4	1.5 - 10.5	0.1 ± 0.4	0.9 - 2.27	5.7
Cirrholovenia polynema Kramp, 1959	-	-	0.1 ± 0.2	0.4 - 1.07	4.6
Aequorea spp.	-	-	0.1 ± 0.2	0.9 - 1.2	3.4
Cunina frugifera Kramp, 1948	-	-	0.1 ± 0.1	1 - 2.7	3.4
Helgicirrha angelicae Tosetto, Neumann-Leitão & Nogueira Junior, 2020	9.6 ± 39.3	4.8 - 176	-	-	3.4
Anthomedusa sp.[illegible]	-	-	0.1 ± 0.6	1.7 - 4.7	2.3
Anthomedusa sp.3	-	-	0.1 ± 0.7	1.9 - 5.1	2.3

Species	Neritic		Oceanic		f
	Mean ± SD	Range of non-zero abundances	Mean ± SD	Range of non-zero abundances	
Eucheilota spp.	0.1 ± 0.14	0.6 - 0.6	0.1 ± 0.2	1.9 - 1.9	2.3
Eutima marajoara Tosetto, Neumann-Leitão & Nogueira Junior, 2020	13.4 ± 45.5	76.4 - 192.6	-	-	2.3
Levenellidae sp	-	-	0.1 ± 0.2	0.5 - 1.7	2.3
Malagazzia carolinae (Mayer, 1900)	0.2 ± 0.8	0.8 - 3.6	-	-	2.3
Bougainvilliidae sp	2.9 ± 10.0	14.9 - 43.1	-	-	2.3
Obelia sp.	3.1 ± 13.8	0.6 - 61.8	-	-	2.3
Pegantha martagon Haeckel, 1879	-	-	0.1 ± 0.1	0.4 - 0.6	2.3
Aequorea forskalea Péron & Lesueur, 1810	-	-	0.1 ± 0.2	1.9 - 1.9	1.1
Stomotoca atra L. Agassiz, 1862	0.1 ± 0.2	0.8 - 0.8	-	-	1.1
Anthomedusa sp.2	-	-	0.1 ± 0.2	1.9 - 1.9	1.1
Anthomedusa sp.4	-	-	0.1 ± 0.3	2.2 - 2.2	1.1
Bougainvillia muscus (Allman, 1863)	-	-	0.1 ± 0.1	0.5 - 0.5	1.1
Campanulariidae sp.	-	-	0.1 ± 0.3	2.5 - 2.5	1.1
Corynidae sp.	-	-	0.1 ± 0.1	0.5 - 0.5	1.1
Cytaeis sp.2	-	-	0.1 ± 0.1	0.9 - 0.9	1.1
Eirene lactea (Mayer, 1900)	0.2 ± 0.8	3.5 - 3.5	-	-	1.1
Hydromedusae sp.	-	-	0.1 ± 0.1	0.6 - 0.6	1.1
Laodicea undulata (Forbes & Goodsir, 1853)	1.1 ± 5.1	22.9 - 22.9	-	-	1.1
Mitrocomium cirratum Haeckel, 1879	0.1 ± 0.4	1.6 - 1.6	-	-	1.1
Octophialucium bigelowi Kramp, 1955	0.2 ± 0.7	3.2 - 3.2	-	-	1.1
Octophialucium haeckeli (Vannucci & Soares Moreira, 1966)	0.2 ± 0.8	3.5 - 3.5	-	-	1.1
Olindias sp.	0.1 ± 0.4	1.59 - 1.6	-	-	1.1
Pegantha triloba Haeckel, 1879	-	-	0.1 ± 0.3	2.8 - 2.8	1.1
**Scyphomedusae**					

**Species**	**Neritic**		**Oceanic**		**f**
	**Mean ± SD**	**Range of non-zero abundances**	**Mean ± SD**	**Range of non-zero abundances**	
Nausithoe punctata Kölliker, 1853	0.6 ± 2.3	1.49 - 10.3	0.3 ± 0.7	0.3 - 3.3	20.7
Nausithoe maculata Jarms, 1990	-	-	0.1 ± 0.2	1.7 - 1.7	1.1
**Other**					
Cerinula larvae	8.9 ± 19.7	1.49 - 82.6	0.4 ± 0.9	0.2 - 4.6	29.9
Ephyrae larvae	0.5 ± 1.5	1.43 - 6.1	0.1 ± 0.3	0.4 - 1.8	8
Athorybia larvae	-	-	0.1 ± 0.6	0.3 - 4.9	2

Liriope tetraphylla also dominated in abundance, representing 46% of all medusae. Other representative medusae were *Persa incolorata* (28.8%) and *A. hemistoma* (15.6%). Among siphonophores, *Enneagonum hyalinum* was the most abundant (19.1%), followed by *D. bojani* (18.2%), *C. appendiculata* (12.5%), *E. mitra* (10.8%) and *Muggiaea kochii* (10.6%).

3.3. Spatial distribution patterns

Total medusa abundance was higher and more variable over the continental shelf, ranging from 1.4 to 1710 ind. 100 m^{-3} (891.7±1161.3 in average). In this province, while medusa species richness was higher in the stations influenced by the ARP, high abundances occurred in stations both inside and outside of the ARP. In oceanic waters, highest medusa abundance occurred at stations located in the area influenced by the ARP and species richness was similar both inside and outside the influence of ARP (Fig. 4).

Figure 4. Geographic distribution of number of species and total abundance of hydromedusae and siphonophores found in the first 200 m of the water column (mean of 120 and 300 µm meshes) in October 2012.

Although widespread all over the sampled area, the hydromedusa *L. tetraphylla* dominated, with significant higher abundances (Fig. 5, Table 2) in the neritic stations outside the ARP. Its presence was also constant, although less abundant, in the retroflection area and in the NECC. Lower catches occurred in most oceanic stations under the influence of the NBC. *P. incolorata*, *Helgicirrha angelicae* and *Cunina octonaria* occurred in large abundances and almost exclusively in neritic stations under influence of the ARP. *Eutima marajoara* occurred in high abundance and exclusively at station 1, inside the estuary. *A. hemistoma* was the dominant medusa through most of the oceanic habitat. *S. eurygaster* was also representative in the open

ocean, usually in lower densities. *Cytaies* sp. 1 and *Solmundella bitentaculata* occurred scattered in low abundances all over the area (Fig. 5).

Figure 5. Geographic distribution and abundance of the dominant hydromedusae found in the first 200 m of the water column (mean of 120 and 300 µm meshes) in October 2012. Species are sorted according to total abundance.

Siphonophores species richness and overall abundance were more constant and typically high in oceanic stations, where it averaged 19.1±3.8 species and 193.8±107.7 ind. 100 m^{-3}, respectively. Differently, over the continental shelf, siphonophore species richness was considerably lower, typically <5 (3.6±3.4 in average), and they were absent at stations 2 and 4. Abundances in neritic stations also tended to be lower, but very high abundance (up to 3381.3 ind. 100 m^{-3}) occurred at station 9 (Fig. 4).

Enneagonum hyalinum and *M. kochii* occurred in high abundance and almost exclusively in neritic stations under the influence of the ARP. Although widespread and abundant all over the area, *D. bojani* abundance was significantly higher in the oceanic stations under the influence of the ARP (Fig. 6, Table 2). Also widespread, but with lower densities, *Diphyes dispar and N. bijuga* were more abundant in stations under the influence of the ARP or near its boundaries both in the neritic (significant for both species) and oceanic habitats (significant for *N. bijuga*, Fig. 6, Table 2). All other abundant siphonophores occurred exclusively at oceanic stations and the neritic station 45, located near the shelf break (Fig. 6). No clear spatial patterns were observed in the proportion of eudoxid and polygastric stages of calycophoran siphonophores.

Figure 6. Geographic distribution and abundance of the dominant siphonophores found in the first 200 m of the water column (mean of 120 and 300 µm meshes) in October 2012. *Except for *Nanomia bijuga.* Species are sorted according to total abundance.

Table 2. Results of the Analysis of Variance testing differences in the abundance of representative planktonic cnidarian species among neritic and oceanic provinces inside (ARP) and outside (Out ARP) the influence of the Amazon River Plume and Tukey post-hoc test. Significant p-values (< 0.05) are in bold. Different Letters indicate significant pair-wise difference among areas in the Tukey test. Species are sorted according to total abundance.

**Species**	**F**	**p**	**Neritic**		**Oceanic**	
			**Out ARP**	**ARP**	**Out ARP**	**ARP**
Liriope tetraphylla	7.92	**0.00**	a	b	c	c
Persa incolorata	311.87	**0.00**	a	b	a	a
Enneagonum hyalinum	318.16	**0.00**	a	b	a	a
Diphyes bojani	15.35	**0.00**	a	a	b	c
Chelophyes appendiculata	19.26	**0.00**	a	a	b	b
Aglaura hemistoma	13.89	**0.00**	a	a	b	b
Eudoxoides mitra	19.07	**0.00**	a	a	b	c
Muggiaea kochii	110.53	**0.00**	a	b	a	a
Diphyes dispar	12.21	**0.00**	a	b	a	a
Bassia bassensis	17.41	**0.00**	a	a	b	b
Eudoxoides spiralis	9.94	**0.00**	a	a	b	c
Sulculeolaria chuni	3.81	**0.02**	a	a	b	b
Abylopsis tetragona	10.93	**0.00**	a	a	b	b
Abylopsis eschscholtzii	5.23	**0.00**	a	a	b	b
Nanomia bijuga	7.68	**0.00**	a	b	c	d

3.4. Assemblage structure

The cluster analysis depicted three main groups with low resemblance to each other (Fig. 7). Group A, with 39.7% similarity, included the neritic stations without influence of the ARP, with the exception of the station 45. The group was represented mainly by *L. tetraphylla* in high abundance (Table 2). The two neritic stations under influence of the ARP belonged to Group

B. With an average similarity of 69.5%, this group was mainly represented *P. incolorata*, *E. hyalinum*, *M. kochii* and *D. dispar* (Table 3).

Figure 7. Cluster analysis dendogram indicating five groups of stations with similar planktonic cnidarian communities in the Western Equatorial Atlantic Ocean.

Table 3. Results of Similarity Percentage analysis (SIMPER), showing the relative contribution of planktonic cnidarian species in the formation of the groups defined in the Cluster analysis.

Species	A	B	C1	C2	C3
Abylopsis eschscholtzi	-	-	5.2	4.2	4.4
Abylopsis tetragona	-	-	11.9	6.9	4.6
Agalma okeni	-	-	-	2.1	2
Aglaura hemistoma	-	-	11.2	9.9	10.7
Bassia bassensis	-	-	17.1	10.1	7.9
Ceratocymba leuckartii	-	-	-	-	2.1
Chelophyes appendiculata	-	-	18.8	13.4	8.2
Cunina octonaria	-	6.8	-	-	-
Diphyes bojani	-	-	10.8	9.9	13.1
Diphyes dispar	8.1	11.5	-	-	-
Enneagonum hyalinum	-	16.3	-	-	-
Eudoxoides mitra	-	-	-	10.8	11.2
Eudoxoides spiralis	-	-	11.3	7.7	-
Cerinula larvae	-	10.1	-	-	-
Lensia campanella	-	-	-	-	2.7
Liriope tetraphylla	84.1	5.7	5	5.2	5.1
Muggiaea kochi	-	14.6	-	-	-
Nanomia bijuga	-	6.7	-	2.4	3.2
Persa incolorata	-	21.3	-	-	-
Rhopalonema velatum	-	-	-	-	2.1
Sminthea eurygaster	-	-	-	1.8	1.9
Solmundella bitentaculata	-	-	-	-	1.6
Sulculeolaria biloba	-	-	-	-	2.6
Sulculeolaria chuni	-	-	-	6.2	4.9
Sulculeolaria turgida	-	-	-	-	2.3

Group C, that occurred mainly in oceanic stations, encompassed three subgroups and two outliers. Stations 45 and 10 were located near the shelf break and considered outliers.

Subgroup C1 clustered stations 34 to 38, located in the southeastern portion of the study area (Fig. 7). It was mainly represented by *C. appendiculata*, *B. bassensis* and *A. tetragona* and differed from other oceanic groups by the low occurrences of *E. mitra* and *S. chuni* (Fig. 6; Table 2). The similarity within the group was 69.2%. Subgroup C2 included the remaining oceanic stations outside the influence of the ARP. With an average similarity of 69.5%, *C. appendiculata*, *E. mitra* and *B. bassensis* were the main representative of this subgroup (Fig.7; Table 3). Except for station 17 and 22 placed in subgroup C2, all oceanic stations under the influence of the ARP and stations 25 and 26 located near its limit were included in subgroup C3 (Fig. 7; Table 3), with 69.3% of similarity. This subgroup was mainly represented by high abundances of *D. bojani, E. mitra* and *A. hemistoma*.

3.5. Responses to mesoscale processes and environmental gradients

The first two axes of the RDA explained 54.9% of planktonic cnidarian species variance (Table 4). Monte Carlo test showed that the first (F-ratio = 18.3, P-value = 0.002) and all canonical axes together (F-ratio = 6.3, P-value = 0.002) were significant. Axis 1 (37.8% of variance) was mainly related to the oceanic/neritic gradient. Zooplankton biomass and cold-core cyclonic eddies were positively related to this axis. The second axis (17% of variance) was negatively related to surface salinity and cyclonic eddies, and positively related to the ARP, fluorescence and zooplankton biomass. Axes 3 and 4 explained together less than 10% of species variance and were not considered (Fig. 8, Table 4).

Figure 8. Redundancy analysis relating dominant planktonic cnidarian species to environmental gradients and mesoscale processes in the Western Equatorial Atlantic Ocean. *L.tet = Liriope tetraphylla, D.boj = Diphyes bojani, A.hem = Aglaura hemistoma, E.mit = Eudoxoides mitra, B.bas = Bassia bassensis, A.tet = Abylopsis tetragona, N.bij = Nanomia bijuga, C.app = Chellophyes appendiculata, A.esc = Abylopsis eschscholtzii, S.chu = Sulculeolaria chuni, D.dis = Diphyes dispar,*

A.oke = *Agalma okeni, E.spi* = *Eudoxoides spiralis, S.eur* = *Sminthea eurygaster, C.leu* = *Ceratocymba leuckartii, S.tur* = *Sulculeolaria turgida, R.vel* = *Rhopalonema velatum, L.cam* = *Lensia campanella, A.ele* = *Agalma elegans, C.sp1* = *Cytaeis* sp. 1, *S.bit* = *Solmundella bitentaculata, S.bil* = *Sulculeolaria biloba, P.inc* = *Persa incolorata, E.hya* = *Enneagonum hyalinum, M.koc* = *Muggiaea kocchii,* ARP = Presence of Amazon River Plume, NBC = North Brazilian Current, NECC = North Equatorial Countercurrent, Eddy + = Anticyclonic eddy, Eddy - = Cyclonic eddy, SST = Sea surface temperature, SSS = Sea surface salinity, DO = Dissolved oxygen, Flu = Fluorescence, Zoo = Zooplankton Biomass.

Table 4. Summary of the Redundancy Analysis (RDA) performed between the cnidarian assemblage and environmental explanatory variables from the Western Equatorial Atlantic Ocean

	**Axis 1**	**Axis 2**
Eigenvalues	0.378	0.17
Species-environment correlations	0.912	0.95
**Cumulative variance (%):**		
Of species data	37.8	54.9
Of species-environment relationships	54.1	78.5
**Correlations of explanatory variables:**		
Neritic province	0.8967	-0.1822
Oceanic province	-0.8967	0.1822
Amazon River Plume	-0.005	0.6031
North Brazil Current	0.4703	-0.3454
North Equatorial Counter Current	-0.4377	0.3145
Anticyclonic eddy	0.0142	0.283
Cyclonic eddy	0.529	-0.476
Sea surface temperature	-0.0872	0.2824
Sea surface salinity	-0.1898	-0.8106
Dissolved Oxygen	-0.4794	0.4573
Fluorescence	0.2873	0.7395
Zooplankton Biomass	0.5882	0.3632

Most oceanic species were closely related to the left portion of the first axis with few relation with axis 2, which represented the ARP and surface salinity gradient, reflecting their wide distribution over the oceanic province and low effect of the ARP on their distribution. Other

oceanic species, such as *D. bojani*, *E. mitra* and *R. velatum* correlated with both the negative portion of axis 1 and the positive portion of axis 2, indicating their higher abundance in the oceanic environment under influence of the ARP (Fig. 8).

Nanomia bijuga, *S. bitentaculata* and *L. campanella* were close related with the positive portion of axis two, indicating their preference for the low salinity environment of the ARP in both oceanic and neritic habitats. *Enneagonum hyalinum*, *M. kochii*, *P. incolorata* and *D. dispar* correlated with the positive portions of axes 1 and 2, reflecting their high abundances in neritic stations under the ARP influence, where the higher primary production and food availability occurred. *Liriope tetraphylla* was positively related to the first axis, as a result of its large abundance over the continental shelf both inside and outside the ARP and in the cold-core cyclonic eddy (Fig. 8).

4. Discussion

Our results show that the Amazon River Plume drives physical and ecological processes that affect the distribution and abundance of planktonic cnidarians both over the continental shelf and in the open ocean in the Western Equatorial Atlantic Ocean. Although our study was focused on cnidarians, analogous effects are expected to the whole planktonic community [e.g. 28]. Over the continental shelf, we observed a strong influence of the ARP [surface salinity <35; ,43] in two stations (8 and 9) to the west of the study domain (Fig. 2c). This is the zone where the NBC is retroflected to the north (Fig 2a), spreading the ARP over the entire length of the shelf [58]. In addition to the freshwater input lowering salinity, the ARP discharges a massive amount of nutrients, organic matter and sediments [24], boosting the primary production (evidenced by the fluorescence; Figs. 2g, 3d). Consequently, higher trophic levels, including planktonic cnidarians benefit from the higher food availability derived from bottom-up processes.

Over the continental shelf, the ARP was restricted to the first 8 m of the water column. Below lied an oxygen minimum layer (Figs. 3 a, e), likely in consequence of the high rates of organic matter that sink and fuel microbial respiration [59]. These features compressed the bulk of productivity in the ARP, both vertically and horizontally (Fig. 9). Although with the oblique trawls performed in our study, we could not infer the exact vertical position of the huge amount of cnidarians observed in the area of influence of the ARP and its relation with the oxygen minimum layer, such three-dimensional habitat configuration seems particularly beneficial for cnidarians. While cnidarians usually are not restricted by oxygen concentration due their low metabolism, low oxygen levels compress the suitable habitat for fish and other predators with higher metabolic demand to the thin surface layer [32,60–62]. In addition, in this thin productive layer the water is turbid due to the sediment runoff and suspended particulate matter. Cnidarians, which are not visual feeders, can take vantage from other competitors and, due their fast reproduction, proliferate in large population blooms [63–65]. Thus, in the habitat shaped by the ARP, we observed the highest abundance of planktonic cnidarians and high species richness for both hydromedusae and siphonophores (cluster group B). Such huge abundances are in general higher than the observed over the continental shelf in other tropical systems along the Western Atlantic [21,66] and the maxima observed in high productive scenarios, such as upwelling and river runoff, in other western boundary systems [20,67].

Figure 9. Schematic representation of the Amazon River Plume (ARP) and associated oxygen minimum zone (OMZ) and effects of habitat structure on planktonic cnidarian assemblages in the Equatorial Western Atlantic Ocean.

Among several species that were abundant over the continental shelf under influence of the ARP, the hydromedusae *P. inclorata*, *C. octonaria* and *H. angelicae,* and the siphonophores *E. hyalinum* and *M. kocchii* occurred almost exclusively within this habitat. *H.*

angelicae was recently described from the study area [68], perhaps being an endemic representative of the ARP fauna. *M. kocchii* and *C. octonaria* are typical coastal water species in the Western Atlantic [5,21,39,69], therefore its absence in the remaining habitats of the study area are expected. Distinctly, *P. inclorata* and *E. hyalinum* have been reported with contrasting niches in different works. In some of them, similar to our results, they were reported as coastal water species [33,35,70,71] and others as high saline oceanic species [30,72]. Such differences can represent intraspecific variability or even cryptic species.

In the portion of the continental shelf outside the influence of the ARP, as a typical western boundary system where strong coastward currents carry oligotrophic oceanic waters over the shelf, we observed high salinity oligotrophic waters all along its extension. Under such conditions, characteristically oceanic species were expected to be present and dominant over the continental shelf [20,21]. Instead, the holoplanktonic hydromedusa *L. tetraphylla* dominated almost alone and with quite high abundance this system (Cluster group A), while only occasional catches of other species were observed. *L. tetraphylla* is abundant and widely distributed in neritic habitats of the Western Atlantic [5,21,39,66,73]. However, unlike our results, most studies reported the species co-occurring with other dominant species in both purely coastal assemblages and/or coastal and open ocean mixed assemblages. The continental shelf outside the Amazon basin is generally wider and more complex, with several eddies (Fig. 2b), internal waves and high tide amplitude, than the remaining Tropical Western Atlantic [19,58,74]. Such characteristics are likely behind the processes differencing the cnidarian assemblage between the Amazonian shelf outside the influence of the ARP and other analogous systems.

In the open ocean we observed the influence of the ARP along the NECC, which is derived from the NBC retroflexion and seasonally spreads the ARP to the Central Atlantic [43]. Distinctly from the continental shelf, in this system we did not observe constrasting differences in surface fluorescence when comparing to the remaining open ocean. However, similar to the continental shelf, we indeed observed an oxygen minimum layer below the ARP waters (50~100

m depth). In accordance, the differences in the oceanic cnidarian assemblage inside and outside the influence of the ARP were less preeminent than the observed over the continental shelf, but still notable. The same species were dominant and a similar number of species was observed in the open ocean inside and outside the influence of the ARP. The dominant species were most siphonophores (*D. bojani*, *C. appendiculata*, *E. mitra*, *B. basensis*, *E. spiralis*, *A. tetragona*, *S. chuni* and *A. schscholtzii*) and the hydromedusa *A. hemistoma*. These species are holoplanktonic, and without a benthic polypoid stages that could require substrata for settlement in their life cycle [75,76], they are able to disperse freely. Thus they are typically present in tropical oceanic waters from the Western Atlantic [21,77]. Apparently, the low salinity (minimum of 32.5) observed inside the oceanic ARP, contrasting to the 36 observed in the surrounding tropical water, did not restricted the distribution of these dominant species inside the ARP. However, since our oblique zooplankton samples were performed from 200 meters depth to surface, and the low salinity ARP waters did not exceed 50 meters depth, species intolerant to lower salinity could be distributed below the ARP. A study considering stratified samples is necessary to clarify this issue properly. In any case, some of these species like *A. hemistoma*, *A. tetragona* and *D. bojani* may be occasionally found inside estuaries in the Western Atlantic in low salinities [78–80].

Despite the absence of exclusive dominant species, differences in species abundance were perceptible between the habitats inside and outside the ARP in the open ocean. *D. bojani*, *E. mitra*, *D. dispar*, *N. bijuga* and *S. eurygaster* were more abundant inside the influence of the ARP. Meanwhile *Chelophyes appendiculata*, *Bassia bassensis*, *Eudoxoides spiralis* and *Abylopsis tetragona* were slightly more abundant outside the ARP influence. Although we did observed differences in fluorescence between both habitats, higher biomass of cyanobacteria [81] and total zooplankton (Fig. 2h) were observed along the oceanic region under influence of the ARP. Such differences along the food web, the habitat compression caused by the oxygen minimum

zone bellow the ARP and the reduced salinity (Fig. 9) are likely responsible for the differences we observed in the planktonic cnidarian assemblage abundance in the open ocean.

This was the first detaile[illegible]urvey on the cnidarian assemblage structure from a major river plume reaching mesoscale dimensions in neritic and oceanic provinces. Our results showed that the freshwater, nutrients and sediment runoff of the Amazon River shapes habitats with a thin surface highly productive system compressed by a deeper oxygen minimum zone both over the continental shelf and in the open ocean in the Western Equatorial Atlantic Ocean (Fig. 9). We conclude that such habitat structure is particularly advantageous to planktonic cnidarians, which have low metabolic rates and are able to survive in hypoxic zones, resulting in high species richness and abundance. Over the continental shelf, distinctions were sharp and the area under the influence of the ARP presented a diverse assemblage occurring in large abundance. *Enneagonum hyalinum, Muggiaea kochi, Persa incolorata, Cunina octonaria, Diphyes dispar* and *Nanomia bijuga* were representive species in these waters. Outside the ARP, *Liriope tetraphylla* dominated almost alone the neritic habitat. Divergences in the oceanic province were less pronounced but still expressive, occurring mainly in the abundance of dominant species. *Chelophyes appendiculata*, *Bassia bassensis*, *Eudoxoides spiralis* and *Abylopsis tetragona* were slightly more abundant in oceanic areas outside the ARP influence and *Diphyes bojani, Eudoxoides mitra, Diphyes dispar* and *Nanomia bijuga* were more abundant under its influence. As expected, the Western Equatorial Atlantic Ocean under the influence of the ARP revealed a complex system, with many physical and biogeochemical processes occurring simultaneously. This complexity was reflected in the structure of cnidarian assemblage.

Acknowledgements

We are grateful to the officers, crew and scientific team of the Camadas Finas III and Amadeus II research project for their contribution to the success of the operations. The present study was not possible without the support of all members from LABZOO and other laboratories

from UFPE and UFRPE. We are grateful to the Brazilian National Institute of Science and Technology for Tropical Marine Environments (INCT AmbTropic), the Brazilian Research Network on Global Climate Change (Rede CLIMA) and European Integrated CARBOCHANGE for funding the Camadas Finas III survey and to all the scientific team on board. We thank the CNPq (Brazilian National Council for Scientific and Technological Development), which provided a PhD scholarship to E.G.T. (grant 140897/2017-8) and a Research Scholarship to S.N.L. and M.A. This work is a contribution to the LMI TAPIOCA (www.tapioca.ird.fr), CAPES/COFECUB program (88881.142689/2017-01), the European Union's Horizon 2020 projects PADDLE (grant agreement No. 73427) and TRIATLAS (grant agreement No. 817578).

Declarations

Conflict of interest. The authors declare no competing interests.

Ethics approval. All applicable international, national, and/or institutional guidelines for care and use of animals were followed by the authors.

Sampling and field studies. All necessary permits for sampling and observational field studies have been obtained by the authors from the competent authorities and are mentioned in the acknowledgements.

Literature cited

1. Graham WM, Pagès F, Hamner WM. A physical context for gelatinous zooplankton aggregations: a review. Hydrobiologia. 2001;451: 199–2012.

2. García-Comas C, Stemmann L, Ibanez F, Berline L, Mazzocchi MG, Gasparini S, et al. Zooplankton long-term changes in the NW Mediterranean Sea: Decadal periodicity forced by winter hydrographic conditions related to large-scale atmospheric changes? J Mar Syst. 2011;87: 216–226. doi:10.1016/j.jmarsys.2011.04.003

3. Brotz L, Cheung WWL, Kleisner K, Pakhomov E, Pauly D. Increasing jellyfish populations: trends in Large Marine Ecosystems. Hydrobiologia. 2012;690: 3–20. doi:10.1007/s10750-012-1039-7

4. Pagès F, Gili. Effects of large-scale advective processes on gelatinous zooplankton populations in the northern Benguela ecosystem. Mar Ecol Prog Ser. 1991;75: 205–215.

5. Nogueira Júnior M, Brandini FP, Codina JCU. Distribution of planktonic cnidarians in response to South Atlantic Central Water intrusion in the South Brazilian Bight. Cont Shelf Res. 2014;89: 93–102. doi:10.1016/j.csr.2014.02.022

6. Boero F, Brotz L, Gibbons MJ, Piraino S, Zampardi S. 3.10 Impacts and effects of ocean warming on jellyfish. In: Laffoley D, Baxter JM, editors. Explaining Ocean Warming: Causes, Scale, Effects and Consequences. Switzerland: UICN; 2016. pp. 213–237.

7. Guerrero E, Marrodán A, Sabatés A, Orejas C, Gili JM. High spatial heterogeneity of two planktonic cnidarian species related to the variability of a shelf-slope front at short time scales. Sci Mar. 2016;80: 487–497. doi:10.3989/scimar.04452.03A

8. Guerrero E, Gili J-M, Maynou F, Sabatés A. Diversity and mesoscale spatial changes in the planktonic cnidarian community under extreme warm summer conditions. J Plankton Res. 2018;0: 1–19. doi:10.1093/plankt/fby001

9. Mańko MK, Gluchowska M, Weydmann-Zwolicka A. Footprints of Atlantification in the vertical distribution and diversity of gelatinous zooplankton in the Fram Strait (Arctic Ocean). Prog Oceanogr. 2020;189: 102414.

10. Gili JM, Pagès F, Sabatès A, Ros JD. Small-scale distribution of a cnidarian population in the western Mediterranean. J Plankton Res. 1988;10: 385–401. doi:10.1093/plankt/10.3.385

11. Gibbons MJ, Buecher E. Short-term variability in the assemblage of medusae and ctenophores following upwelling events in the southern Benguela ecosystem. Mar Ecol Prog Ser. 2001;220: 169–177. doi:10.3354/meps220169

12. Luo JY, Grassian B, Tang D, Irisson JO, Greer A, Guigand CC, et al. Environmental drivers of the fine-scale distribution of a gelatinous zooplankton community across a mesoscale front. Mar Ecol Prog Ser. 2014;510: 129–149. doi:10.3354/meps10908

13. Pitt KA, Welsh DT, Condon RH. Influence of jellyfish blooms on carbon, nitrogen and phosphorus cycling and plankton production. Hydrobiologia. 2009;616: 133–149. doi:10.1007/s10750-008-9584-9

14. Purcell JE. Jellyfish and Ctenophore Blooms Coincide with Human Proliferations and Environmental Perturbations. Annu Rev Mar Sci. 2012;4: 209–235. doi:10.1146/annurev-marine-120709-142751

15. Purcell JE, Uye S, Lo W. Anthropogenic causes of jellyfish blooms and their direct consequences for humans: a review. Mar Ecol Prog Ser. 2007;350: 153–174. doi:10.3354/meps07093

16. Roux J-P, van der Lingen CD, Gibbons MJ, Moroff NE, Shannon LJ, Smith AD, et al. Jellyfication of Marine Ecosystems as a Likely Consequence of Overfishing Small Pelagic Fishes: Lessons from the Benguela. Bull Mar Sci. 2013;89: 249–284. doi:10.5343/bms.2011.1145

17. Hays GC, Doyle TK, Houghton JDR. A Paradigm Shift in the Trophic Importance of Jellyfish? Trends Ecol Evol. 2018;33: 874–884. doi:10.1016/j.tree.2018.09.001

18. Johns WE, Lee TN, Beardsley RC, Candela J, Limeburner R, Castro B. Annual Cycle and Variability of the North Brazil Current. J Phys Oceanogr. 1998;28: 103–128.

19. Loder JW, Boicourt WC, Simpson JH. Western ocean boundary shelves coastal segment (W). The Sea. 1998;11: 3–27.

20. Thibault-Botha D, Lutjeharms JRE, Gibbons MJ. Siphonophore assemblages along the east coast of South Africa; mesoscale distribution and temporal variations. J Plankton Res. 2004;26: 1115–1128. doi:10.1093/plankt/fbh104

21. Tosetto EG, Bertrand A, Neumann-Leitão S, Costa da Silva A, Nogueira Júnior M. Spatial patterns in planktonic cnidarian distribution in the western boundary current system of the tropical South Atlantic Ocean. Irigoien X, editor. J Plankton Res. 2021;43: 270–287. doi:10.1093/plankt/fbaa066

22. Lentz SJ. The Amazon River plume during AmasSeds: subtidal current variability and the importance of wind forcing. J Geophys Res. 1995;100: 2377–2390.

23. Dagg M, Benner R, Lohrenz S, Lawrence D. Transformation of dissolved and particulate materials on continental shelves influenced by large rivers: plume processes. Cont Shelf Res. 2004;24: 833–858. doi:10.1016/j.csr.2004.02.003

24. Signorini SR, Murtugudde RG, McClain CR, Christian JR, Picaut J, Busalacchi AJ. Biological and physical signatures in the tropical and subtropical Atlantic. J Geophys Res Oceans. 1999;104: 18367–18382. doi:10.1029/1999JC900134

25. Hellweger FL, Gordon AL. Tracing Amazon River water into the Caribbean Sea. J Mar Res. 2002;60: 537–549. doi:10.1357/002224002762324202

26. Jo Y-H, Yan XH, Dzwonkowski B, Liu WT. A study of the freshwater discharge from the Amazon River into the tropical Atlantic using multi-sensor data. Geophys Res Lett. 2005;32: 1–4. doi:10.1029/2004GL021840

27. Smith WO, Demaster DJ. Phytoplankton biomass and productivity in the Amazon River plume: correlation with seasonal river discharge. Cont Shelf Res. 1996;16: 291–319. doi:10.1016/0278-4343(95)00007-N

28. Santana CS, Lira SM de A, Varona HL, Neumann-Leitão S, Araujo M, Schwamborn R. Amazon river plume influence on planktonic decapods in the tropical Atlantic. J Mar Syst. 2020;212: 103428. doi:10.1016/j.jmarsys.2020.103428

29. Pagès F. Mesoscale coupling between planktonic cnidarian distribution and water masses during a temporal transition between active upwelling and abatement in the northern Benguela system. South Afr J Mar Sci. 1992;12: 41–52. doi:10.2989/02577619209504689

30. Hosia A, Stemmann L, Youngbluth M. Distribution of net-collected planktonic cnidarians along the northern Mid-Atlantic Ridge and their associations with the main water masses. Deep Sea Res Part II Top Stud Oceanogr. 2008;55: 106–118. doi:10.1016/j.dsr2.2007.09.007

31. Blair NE, Aller RC. Anaerobic methane oxidation on the Amazon shelf. Geochim Cosmochim Acta. 1995;59: 3707–3715.

32. Moura RL, Amado-Filho GM, Moraes FC, Brasileiro PS, Salomon PS, Mahiques MM, et al. An extensive reef system at the Amazon River mouth. Sci Adv. 2016;2: 1–11. doi:10.1126/sciadv.1501252

33. Morales-Ramírez A, Nowaczyk J. El zooplancton gelatinoso del Golfo Dulce, Pacífico de Costa Rica, durante la transición de la estación lluviosa a la seca 1997-1998. Rev Biol Trop. 2006;54: 201–223.

34. Loman-Ramos L, Ordóñez-López U, Segura-Puertas L. Spatial variation of medusan community (Cnidaria) in the Southern Gulf of Mexico, during autumn 1999. Hidrobiológica. 2007;17: 203–212.

35. Sanvicente-Añorve L, Alba C, Alatorre MA, Flores-Coto C. Cross-shelf and vertical distribution of siphonophore assemblages under the influence of freshwater outflows in the southern Gulf of Mexico. Hydrobiologia. 2007;586: 69–78. doi:10.1007/s10750-006-0492-6

36. Andrade C. Distribución estacional y ecología de las medusas (cnidarias: hydrozoa) en la zona costera sur de la bahía de santa elena durante el período (octubre 2004 – octubre 2005). Acta Ocean Pac. 2012;17: 127–138.

37. Andrade C. Sifonoforos (cnidaria, hydrozoa) de aguas superficiales alrededor de la isla santa clara, durante Septiembre y Noviembre del 2007. Acta Ocean Pac. 2012;17: 139–146.

38. Vansteenbrugge L, Van Regenmortel T, De Troch M, Vincx M, Hostens K. Gelatinous zooplankton in the Belgian part of the North Sea and the adjacent Schelde estuary: Spatio-temporal distribution patterns and population dynamics. J Sea Res. 2015;97: 28–39. doi:10.1016/j.seares.2014.12.008

39. Nagata RM, Nogueira Júnior M, Brandini FP, Haddad MA. Spatial and temporal variation of planktonic cnidarian density in subtropical waters of the Southern Brazilian Bight. J Mar Biol Assoc U K. 2014;94: 1387–1400. doi:10.1017/S0025315414000617

40. Coutinho PN. Levantamento do estado da arte da pesquisa dos recursos vivos marinhos do Brasil. Programa REVIZEE. Brasilia: Ministério do Meio Ambiente, dos Recursos Hídricos e da Amazônia Legal.; 1995.

41. Nittrouer CA, DeMaster DJ. The Amazon shelf setting: tropical, energetic, and influenced by a large river. Cont Shelf Res. 1996;16: 553–573. doi:10.1016/0278-4343(95)00069-0

42. Geyer WR, Beardsley RC, Lentz SJ, Candela J, Limeburner R, Johns WE, et al. Physical oceanography of the Amazon shelf. Cont Shelf Res. 1996;16: 575–616. doi:10.1016/0278-4343(95)00051-8

43. Molleri GSF, Novo EML de M, Kampel M. Space-time variability of the Amazon River plume based on satellite ocean color. Cont Shelf Res. 2010;30: 342–352. doi:10.1016/j.csr.2009.11.015

44. Dimoune DM, Birol F, Hernandez F, Léger F, Araujo M. Revisiting the tropical Atlantic western boundary circulation from a 25-year time series of satellite altimetry data. EGUsphere Prepr. 2022; 1–42.

45. Coles VJ, Brooks MT, Hopkins J, Stukel MR, Yager PL, Hood RR. The pathways and properties of the Amazon River Plume in the tropical North Atlantic Ocean: AMAZON RIVER PLUME. J Geophys Res Oceans. 2013;118: 6894–6913. doi:10.1002/2013JC008981

46. Bruto L, Araujo M, Noriega C, Veleda D, Lefèvre N. Variability of CO 2 fugacity at the western edge of the tropical Atlantic Ocean from the 8°N to 38°W PIRATA buoy. Dyn Atmospheres Oceans. 2017;78: 1–13. doi:10.1016/j.dynatmoce.2017.01.003

47. Araujo M, Noriega C, Hounsou-gbo GA, Veleda D, Araujo J, Bruto L, et al. A Synoptic Assessment of the Amazon River-Ocean Continuum during Boreal Autumn: From Physics to Plankton Communities and Carbon Flux. Front Microbiol. 2017;8: 1358. doi:10.3389/fmicb.2017.01358

48. Bouillon J. Hydromedusae. In: Boltovskoy D, editor. South Atlantic zooplankton. Leiden: Backhuys Publishers; 1999. pp. 424–512.

49. Pugh PR. Siphonophorae. In: Boltovskoy D, editor. South Atlantic Zooplankton. Leiden: Backhuys Publishers; 1999. pp. 467–511.

50. Tosetto EG, Neumann-Leitão S, Araujo M, Nogueira Júnior M. Epipelagic cnidarian fauna in the Western Equatorial Atlantic Ocean off the Amazon River Delta. Mar Biodivers. 2022;52: 50. doi:10.1007/s12526-022-01286-0

51. Hosia A, Båmstedt U. Seasonal changes in the gelatinous zooplankton community and hydromedusa abundances in Korsfjord and Fanafjord,western Norway. Mar Ecol Prog Ser. 2007;351: 113–127.

Type text here

52. Pugh PR. The diel migrations and distributions within a mesopelagic community in the North East Atlantic. 7. Siphonophores. Prog Oceanogr. 1984;13: 461–489. doi:10.1016/0079-6611(84)90016-8

53. Omori M, Ikeda T. Methods in marine zooplankton ecology. New York: Willey; 1984.

54. Silva AC, Araújo M, Bourlès B. Variação sazonal da estrutura de massas de água na plataforma continental do Amazonas e área oceânica adjacente. Rev Bras Geofísica. 2005;23: 145–157.

55. Silva AC, Bourles B, Araujo M. Circulation of the thermocline salinity maximum waters off the Northern Brazil as inferred from in situ measurements and numerical results. Ann Geophys. 2009;27: 1861–1873. doi:10.5194/angeo-27-1861-2009

56. Tosetto EG, Neumann-Leitão S, Nogueira Júnior M. Sampling planktonic cnidarians with paired nets: Implications of mesh size on community structure and abundance. Estuar Coast Shelf Sci. 2019;220: 48–53. doi:10.1016/j.ecss.2019.02.027

57. Lepš J, Šmilauer P. Multivariate Analysis of Ecological Data using CANOCO. Cambridge: Cambridge University Press; 2003.

58. Johns WE, Lee TN, Schott FA, Zantopp RJ, Evans RH. The North Brazil Current retroflection: Seasonal structure and eddy variability. J Geophys Res. 1990;95: 22103. doi:10.1029/JC095iC12p22103

59. Diaz RJ, Rosenberg R. Spreading Dead Zones and Consequences for Marine Ecosystems. Science. 2008;321: 926–929. doi:10.1126/science.1156401

60. Bertrand A, Chaigneau A, Peraltilla S, Ledesma J, Graco M, Monetti F, et al. Oxygen: A Fundamental Property Regulating Pelagic Ecosystem Structure in the Coastal Southeastern Tropical Pacific. Slomp CP, editor. PLoS ONE. 2011;6: e29558. doi:10.1371/journal.pone.0029558

61. Hauss H, Christiansen S, Schütte F, Kiko R, Edvam Lima M, Rodrigues E, et al. Dead zone or oasis in the open ocean? Zooplankton distribution and migration in low-oxygen modewater eddies. Biogeosciences. 2016;13: 1977–1989. doi:10.5194/bg-13-1977-2016

62. Lüskow F, Christiansen B, Chi X, Silva P, Neitzel P, Brooks ME, et al. Distribution and biomass of gelatinous zooplankton in relation to an oxygen minimum zone and a shallow seamount in the Eastern Tropical North Atlantic Ocean. Mar Environ Res. 2022;175: 105566. doi:10.1016/j.marenvres.2022.105566

63. Sommer U, Stibor H, Katechakis A, Sommer F, Hansen T. Pelagic food web configurations at different levels of nutrient richness and their implications for the ratio fish production:primary production. Hydrobiologia. 2002;484: 11–20. doi:10.1007/978-94-017-3190-4_2

64. Condon RH, Duarte CM, Pitt KA, Robinson KL, Lucas CH, Sutherland KR, et al. Recurrent jellyfish blooms are a consequence of global oscillations. Proc Natl Acad Sci. 2013;110: 1000–1005. doi:10.1073/pnas.1210920110

65. Schnedler-Meyer NA, Mariani P, Kiørboe T. The global susceptibility of coastal forage fish to competition by large jellyfish. Proc R Soc B Biol Sci. 2016;283: 20161931. doi:10.1098/rspb.2016.1931

66. Suarez-Morales E, Segura-Puertas L, Gasca R. Medusan (Cnidarian) Assemblages off the Caribbean Coast of Mexico. J Coast Res. 1999;15: 140–147.

67. Xu Z, Lin M, Gao Q. Causal analysis of the diversity of medusae in East China Sea. Front Biol China. 2008;3: 300–307. doi:10.1007/s11515-008-0057-3

68. Tosetto EG, Neumann-Leitão S, Nogueira Júnior M. New species of Eirenidae (Hydrozoa: Leptothecata) from the Amazonian coast (northern Brazil). Sci Mar. 2020;84: 421–430. doi:10.3989/scimar.05051.14A

69. Vannucci M. On Brazilian hydromedusae and their distribution in Relation to different water masses. Bol Inst Ocean. 1957;8: 23–109.

70. Gao Q, Chen J, Xu Z, Zhu D. Abundance distribution and seasonal variation of medusae, siphonophores and ctenophores in the Changjiang (Yangtze River) Estuary and the adjacent East China Sea. Acta Ecol Sin. 2015;35: 7328–7337. doi:10.5846/stxb201403210505

71. Gutiérrez-Aguirre MA, Delgado-Blas VH, Cervantes-Martínez A. Diversidad de las hidromedusas (Cnidaria) de la región nerítica del sureste de Tamaulipas, México. Teoría Prax. 2015;11: 153–167. doi:10.22403/UQROOMX/TYP18/06

72. Gasca R. Siphonophores (Cnidaria) and summer mesoscale features in the Gulf of Mexico. Bull Mar Sci. 1999;65: 75–89.

73. Persad G, Hopcroft RR, Webber MK, Roff JC. Abundance, biomass and production of ctenophores and medusae off Kingston, Jamaica. Bull Mar Sci. 2003;73: 379–396.

74. Bai X, Lamb KG, da Silva JC. Small-Scale Topographic Effects on the Generation of Along-Shelf Propagating Internal Solitary Waves on the Amazon Shelf. J Geophys Res Oceans. 2021;126: e2021JC017252.

75. Bouillon J, Gravili C, Pagès F, Gili JM, Boero F, editors. An introduction to Hydrozoa. Paris: Publications Scientifiques du Muséum; 2006.

76. Mapstone GM. Global Diversity and Review of Siphonophorae (Cnidaria: Hydrozoa). Hejnol A, editor. PLoS ONE. 2014;9: 1–37. doi:10.1371/journal.pone.0087737

77. Lo W, Biggs DC. Temporal variability in the night-time distribution of epipelagic siphonophores in the North Atlantic Ocean at Bermuda. J Plankton Res. 1996;18: 923–939. doi:10.1093/plankt/18.6.923

78. Nogueira Júnior M. Gelatinous zooplankton fauna (Cnidaria, Ctenophora and Thaliacea) from Baía da Babitonga (southern Brazil). Zootaxa. 2012;3398: 1–21.

79. Nogueira Júnior M, da Costa BSP, Martinez TA, Brandini FP, Miyashita LK. Diversity of gelatinous zooplankton (Cnidaria, Ctenophora, Chaetognatha and Tunicata) from a subtropical estuarine system, southeast Brazil. Mar Biodivers. 2019;49: 1283–1298. doi:10.1007/s12526-018-0912-7

80. Nogueira M, do Nascimento LS, Maciel PV, Tilbert S, Oliveira LD. Diversity, Species Composition and Assemblage Dynamics of Estuarine Gelatinous and Semi-Gelatinous

Zooplankton from Brazil. In: Hoffmeyer MS, Sabatini ME, Brandini FP, Calliari DL, Santinelli NH, editors. Plankton Ecology of the Southwestern Atlantic. Cham: Springer International Publishing; 2018. pp. 375–412. doi:10.1007/978-3-319-77869-3_18

81. Otsuka AY. Estrutura da comunidade fitoplanctônica na plataforma continental amazônica e região oceânica do Atlântico tropical. Universidade Federal de Pernambuco. 2019.

Figure 1

Click here to access/download;Figure;Fig1.jpg

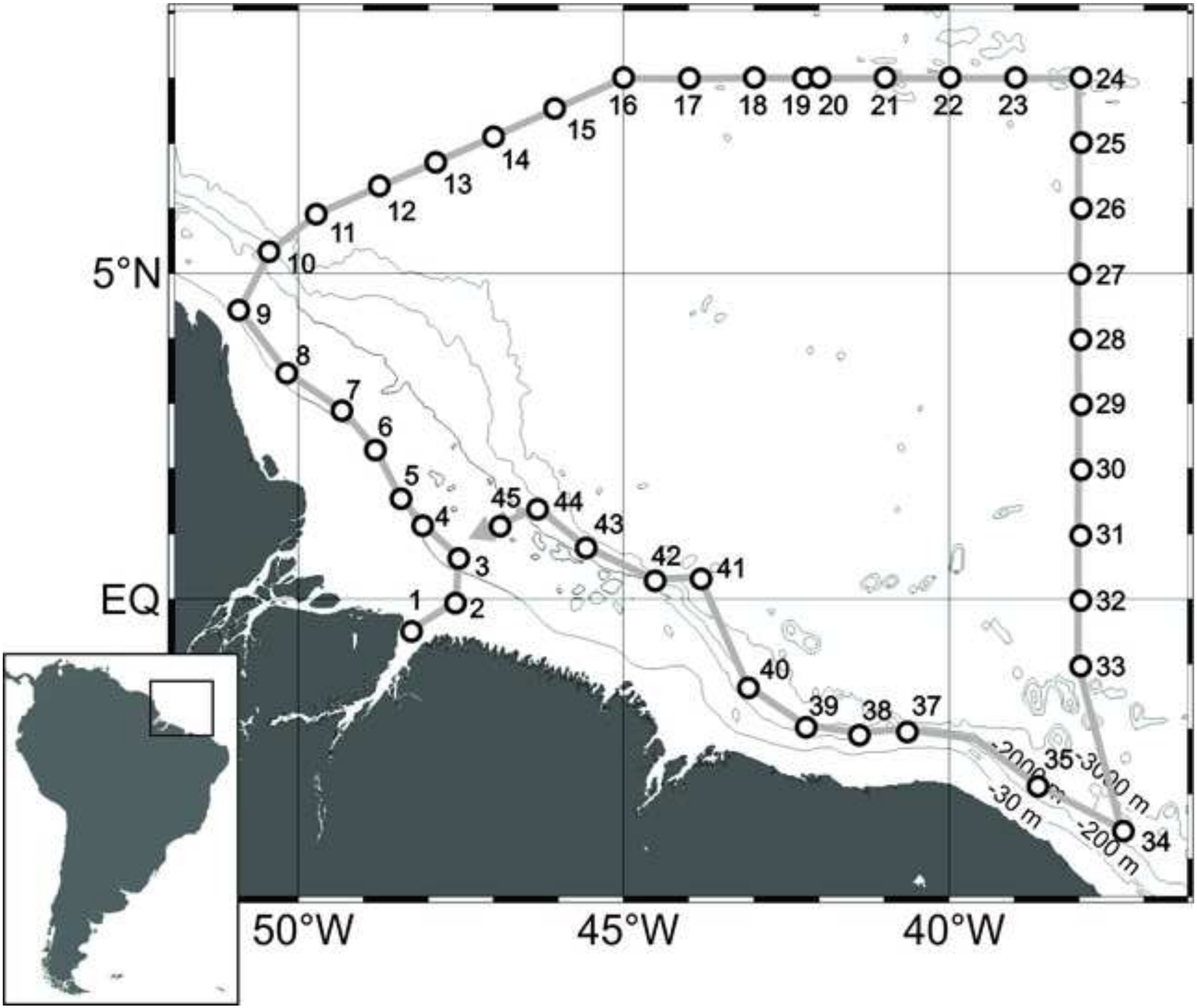

Figure 2

Click here to access/download;Figure;Fig2.jpg

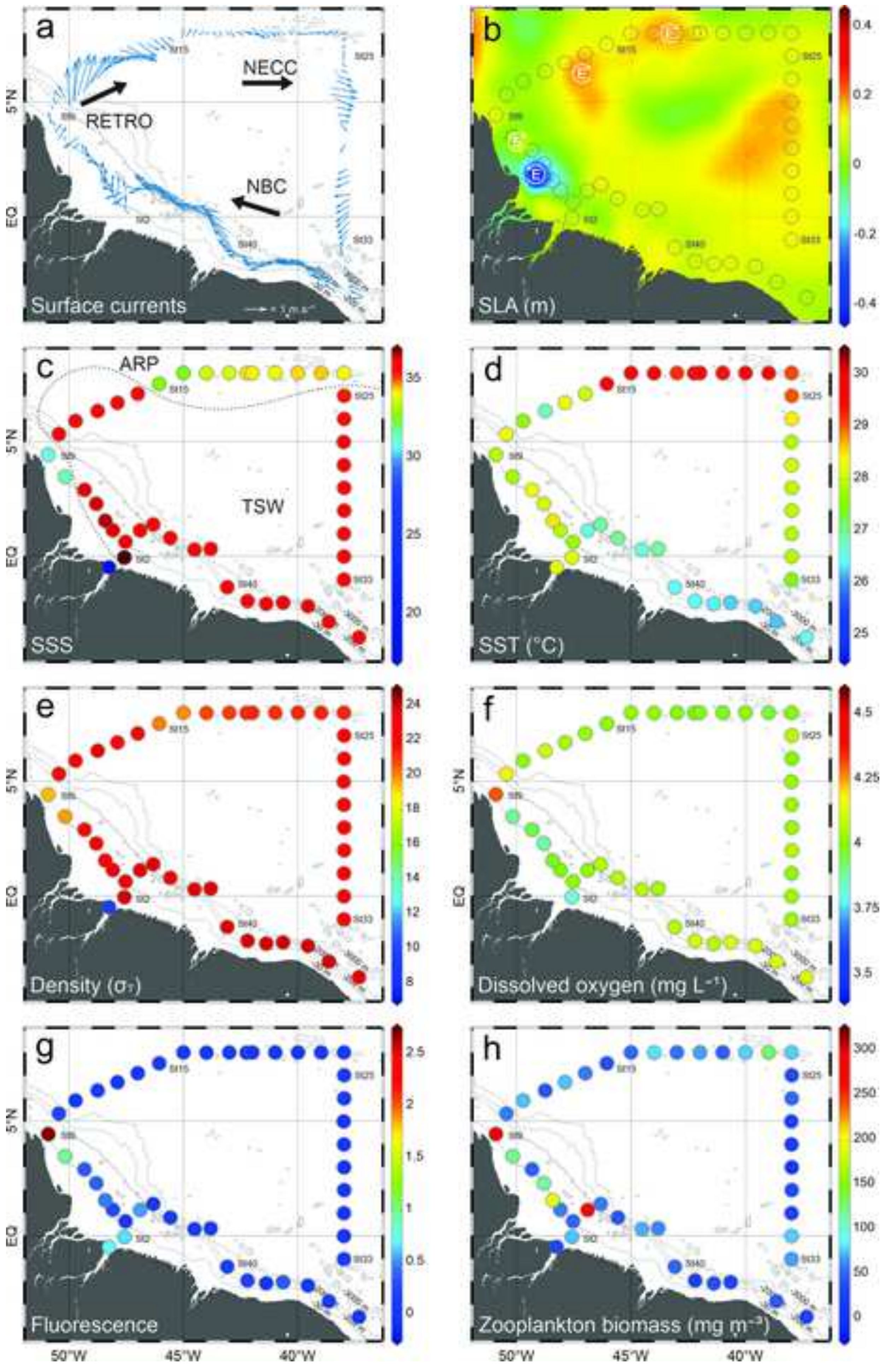

Figure 3

Click here to access/download;Figure;Fig3.jpg

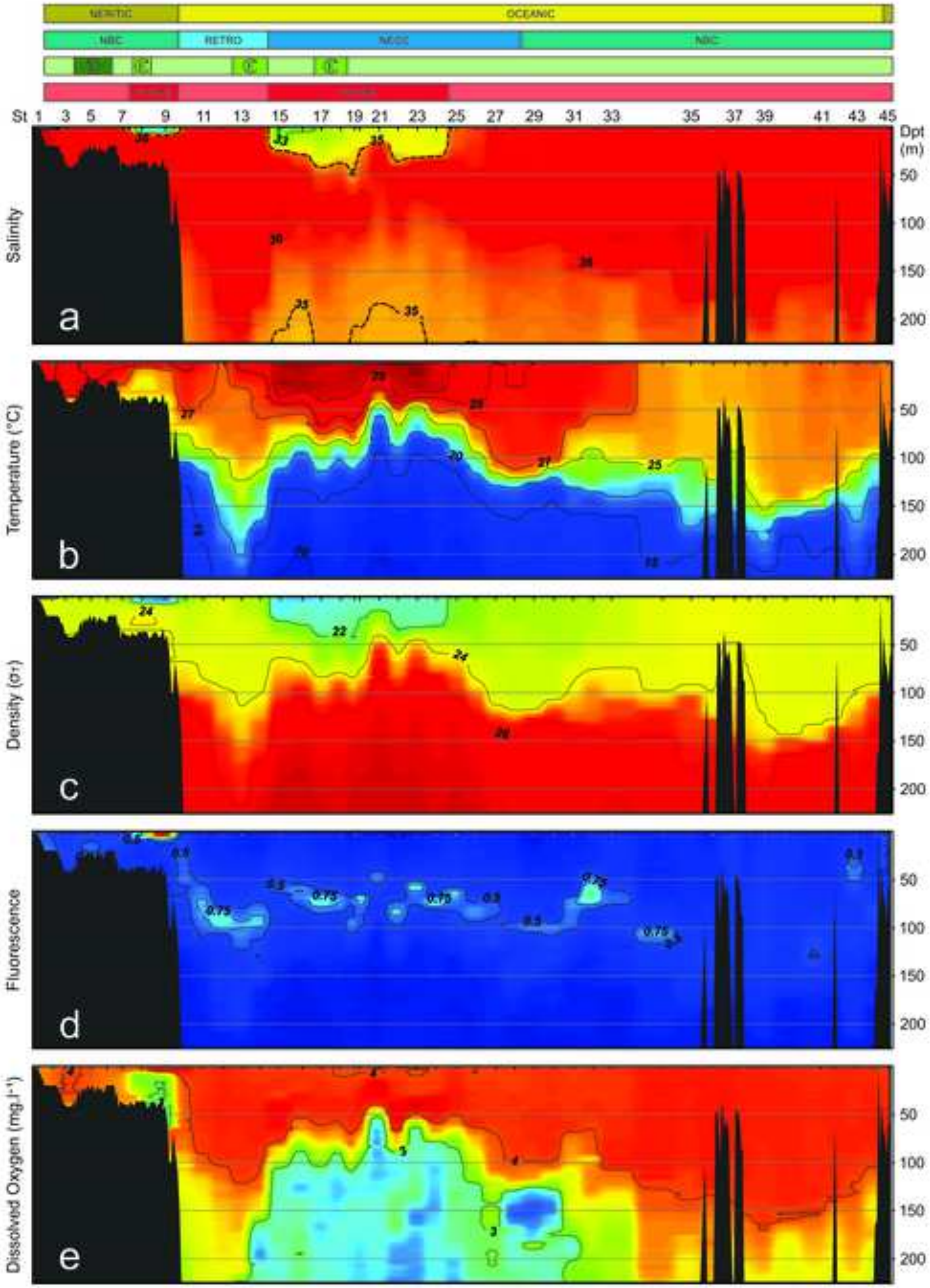

Figure 4

Click here to access/download;Figure;Fig4.jpg

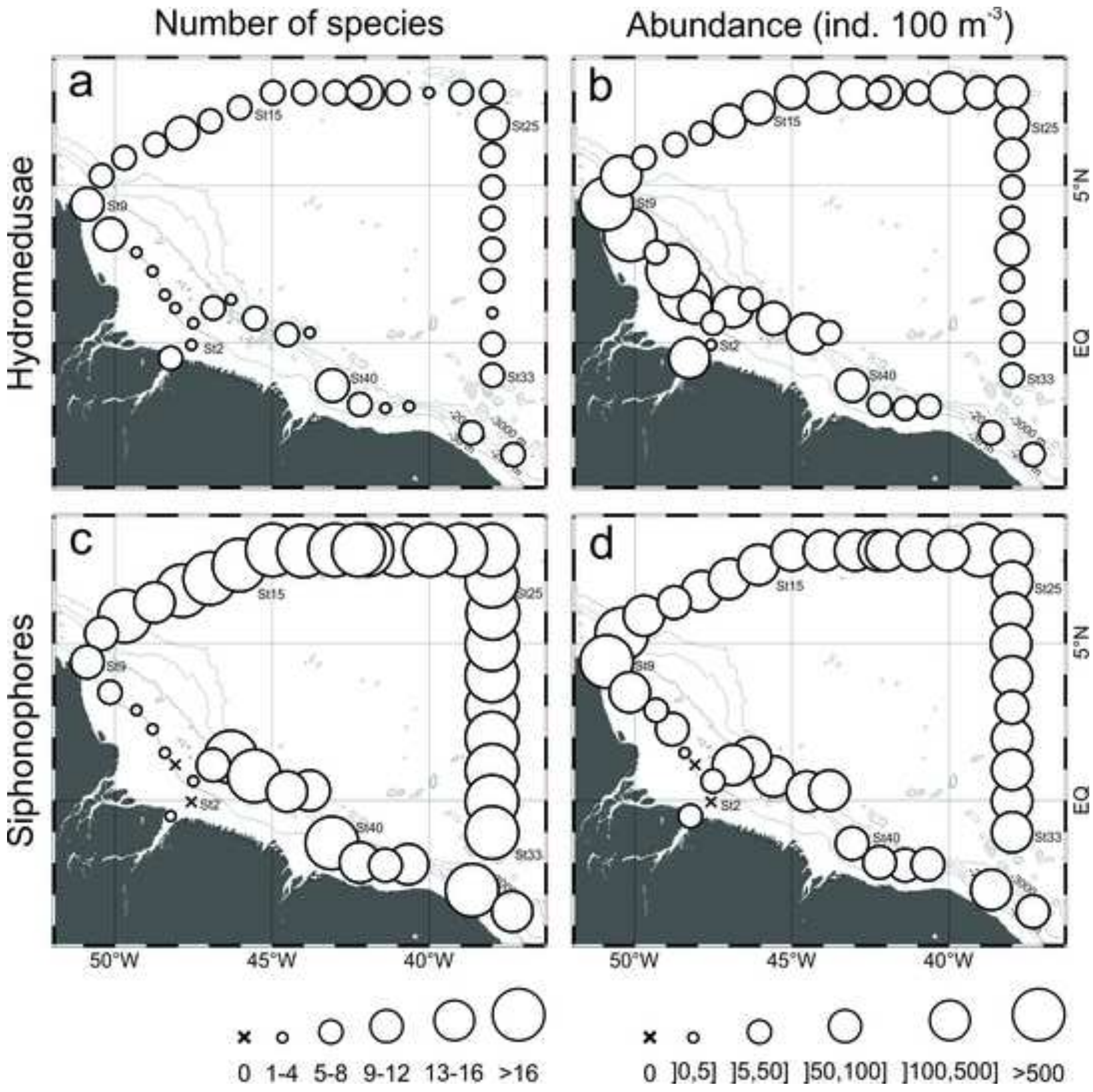

Figure 5

Click here to access/download;Figure;Fig5.jpg

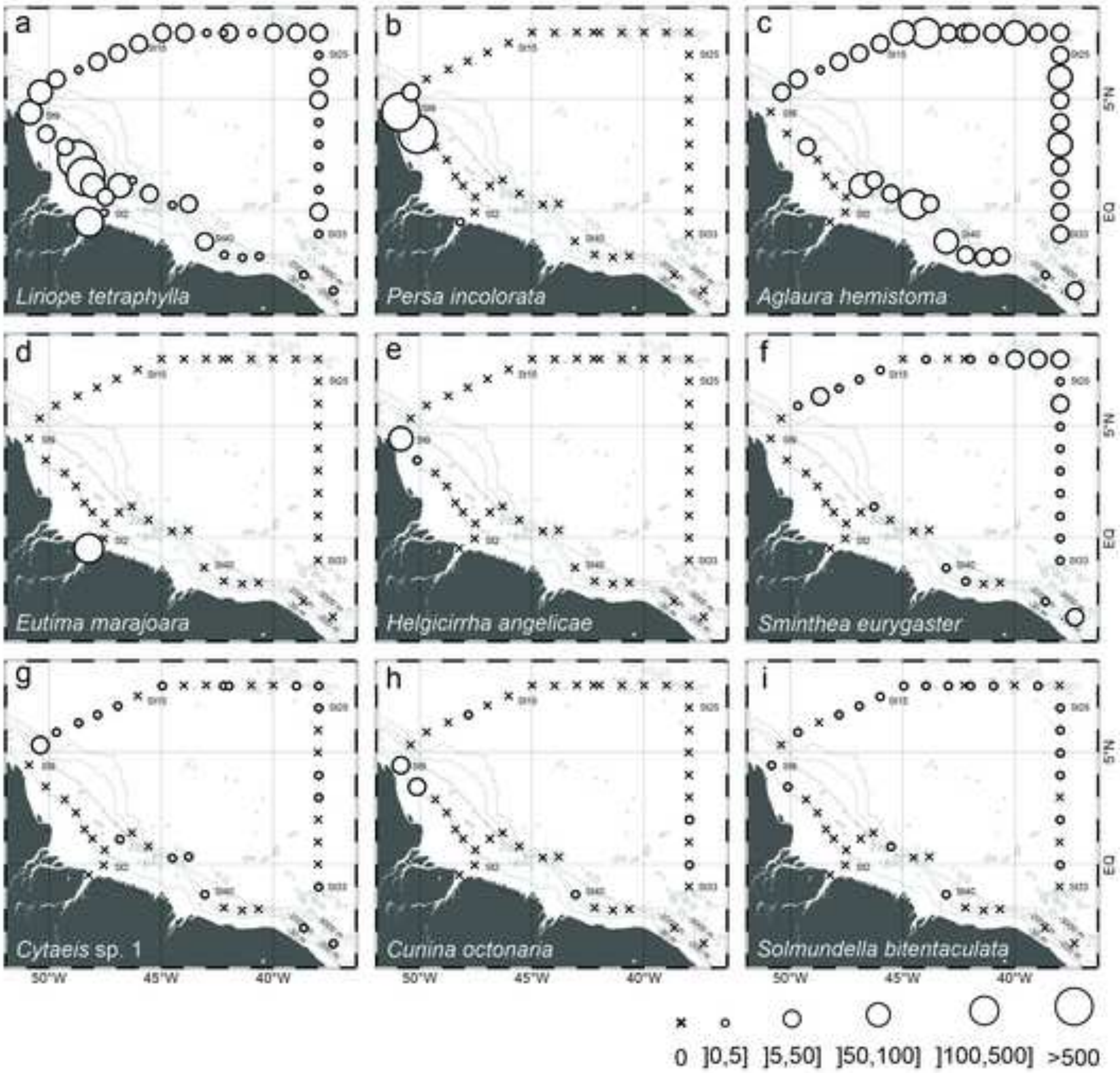

Figure 6

Click here to access/download;Figure;Fig6.jpg

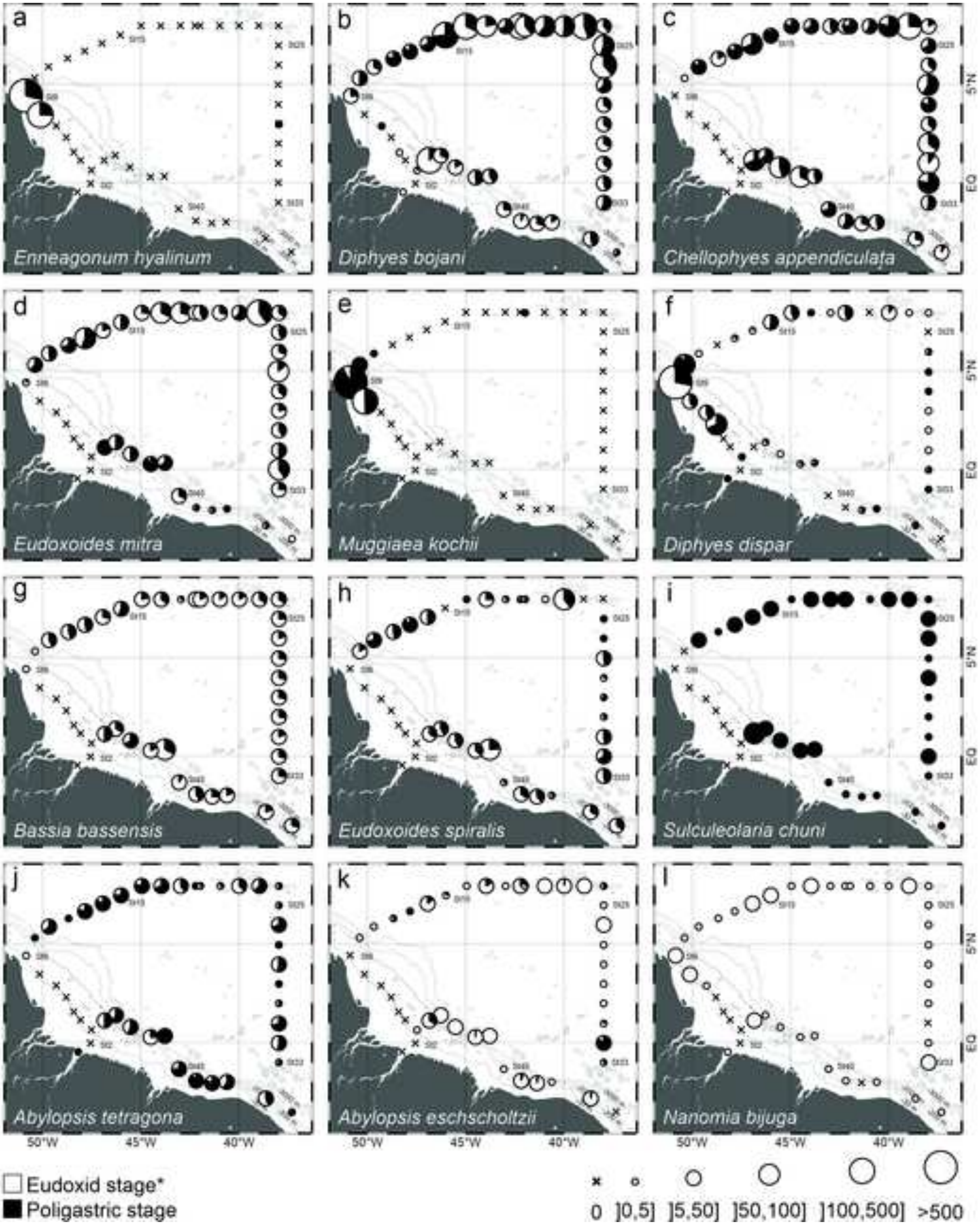

Figure 7

Click here to access/download;Figure;Fig7.jpg

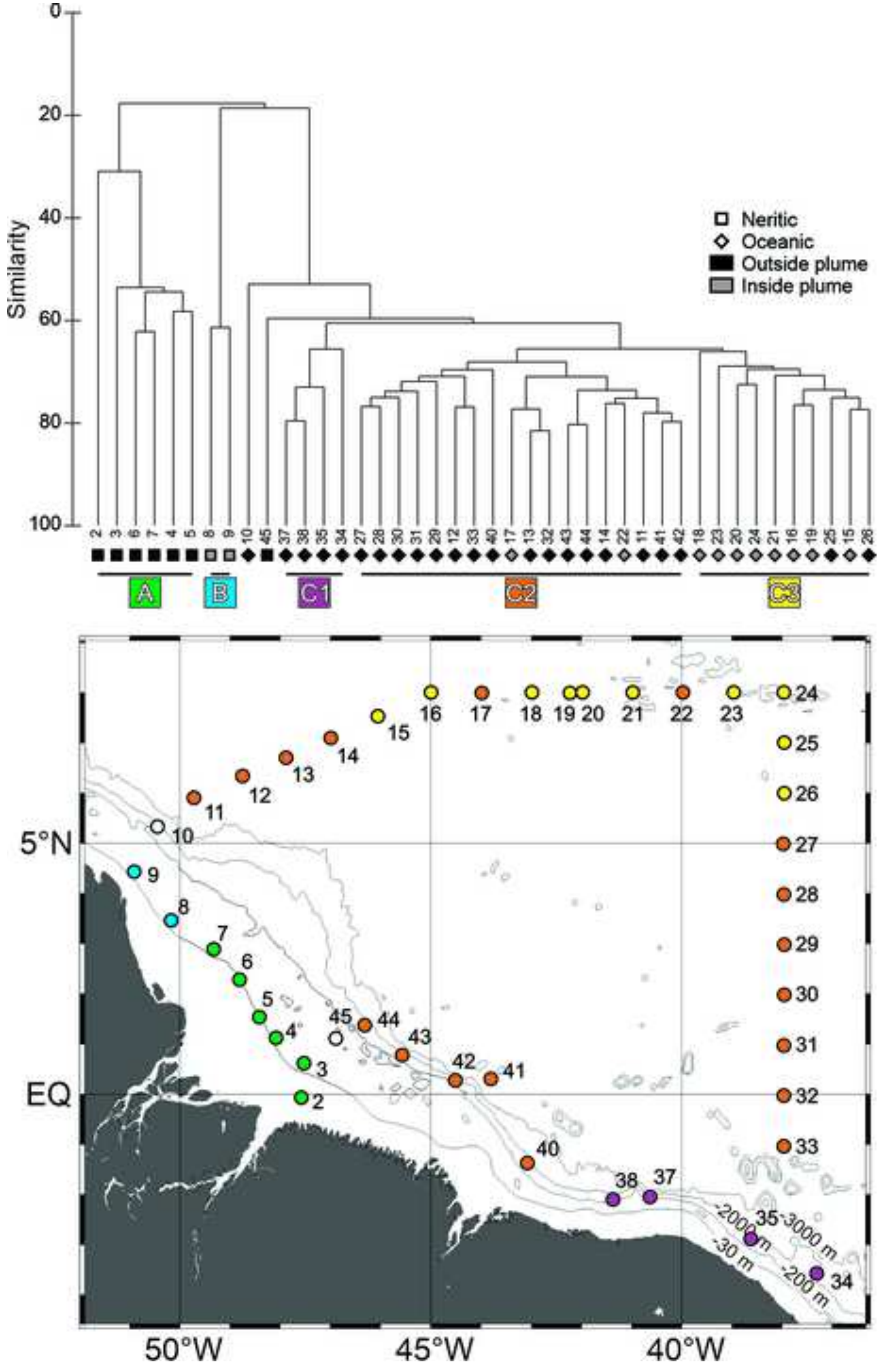

Figure 8

Click here to access/download;Figure;Fig8.jpg

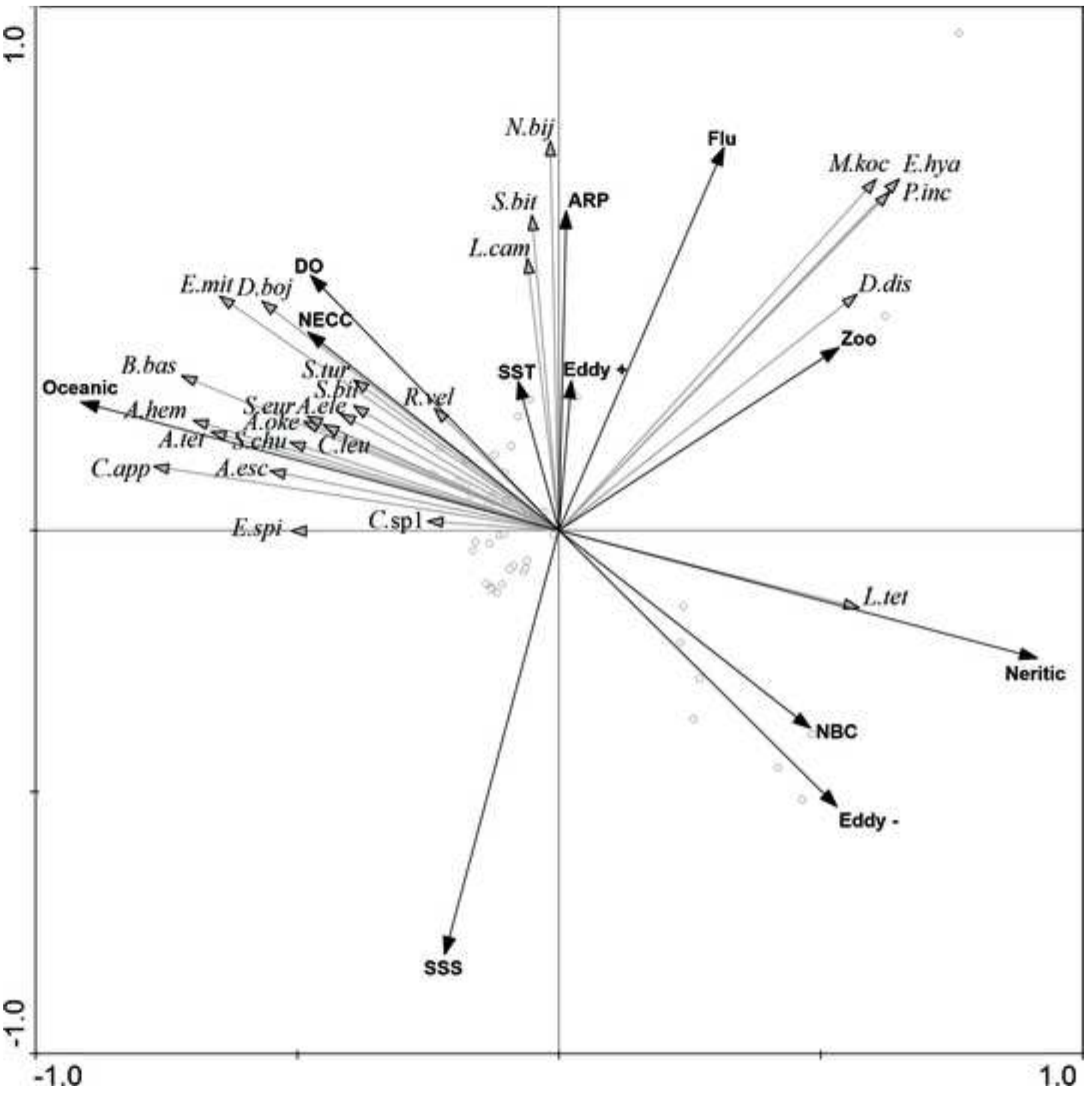

Figure 9

Click here to access/download;Figure;Fig9.jpg

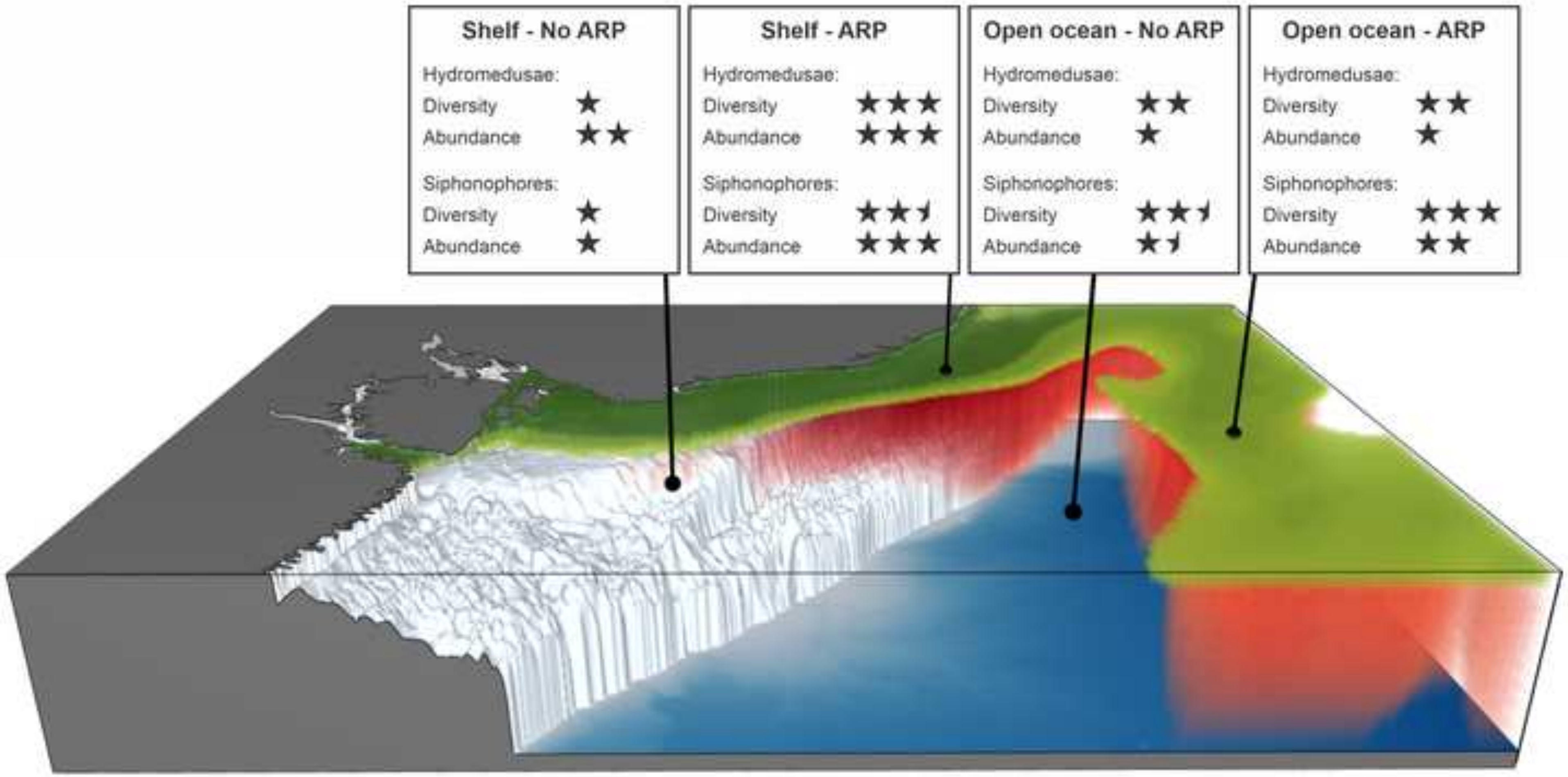

Supplement: Attachment — Submitted filename: PONE-D-23-10398_reviewer.pdf [file pone.0290667.s001.pdf]
